# NNMT‐DNMT1 Axis is Essential for Maintaining Cancer Cell Sensitivity to Oxidative Phosphorylation Inhibition

**DOI:** 10.1002/advs.202202642

**Published:** 2022-11-16

**Authors:** Changqing Wu, Yu'e Liu, Wenju Liu, Tianhui Zou, Shaojuan Lu, Chengjie Zhu, Le He, Jie Chen, Lan Fang, Lin Zou, Ping Wang, Lihong Fan, Hongxiang Wang, Han You, Juxiang Chen, Jing‐Yuan Fang, Cizhong Jiang, Yufeng Shi

**Affiliations:** ^1^ Tongji University Cancer Center Shanghai Tenth People's Hospital of Tongji University School of Medicine Tongji University Shanghai 200092 China; ^2^ Key Laboratory of Spine and Spinal Cord Injury Repair and Regeneration of Ministry of Education Orthopaedic Department of Tongji Hospital Shanghai Key Laboratory of Signaling and Disease Research School of Life Sciences and Technology Tongji University Shanghai 200092 China; ^3^ State Key Laboratory for Oncogenes and Related Genes Key Laboratory of Gastroenterology & Hepatology Ministry of Health Division of Gastroenterology and Hepatology Shanghai Institute of Digestive Disease Renji Hospital School of Medicine Shanghai Jiao Tong University Shanghai 200127 China; ^4^ Clinical Research Unit Children's Hospital of Shanghai Jiaotong University Shanghai 200062 China; ^5^ Department of Respiratory Medicine Shanghai Tenth People's Hospital Tongji University School of Medicine Shanghai 200072 China; ^6^ Department of Neurosurgery Changhai Hospital Naval Medical University NO.168 Changhai Road Shanghai 200433 China; ^7^ Clinical Center for Brain and Spinal Cord Research Tongji University Shanghai 200092 China; ^8^ State Key Laboratory of Cellular Stress Biology Innovation Center for Cell Signaling Network School of Life Sciences Xiamen University Xiamen 361005 China

**Keywords:** biomarker, cancer, DNA methylation, *N*‐methyltransferase (NNMT), oxidative phosphorylation (OXPHOS), S‐adenosyl methionine (SAM)

## Abstract

Lacking a clear understanding of the molecular mechanism determining cancer cell sensitivity to oxidative phosphorylation (OXPHOS) inhibition limits the development of OXPHOS‐targeting cancer treatment. Here, cancer cell lines sensitive or resistant to OXPHOS inhibition are identified by screening. OXPHOS inhibition‐sensitive cancer cells possess increased OXPHOS activity and silenced nicotinamide *N*‐methyltransferase (NNMT) expression. NNMT expression negatively correlates with OXPHOS inhibition sensitivity and functionally downregulates the intracellular levels of S‐adenosyl methionine (SAM). Expression of DNA methyltransferase 1 (DNMT1), a SAM consumer, positively correlates with OXPHOS inhibition sensitivity. NNMT overexpression and DNMT1 inhibition render OXPHOS inhibition‐sensitive cancer cells resistant. Importantly, treatments of OXPHOS inhibitors (Gboxin and Berberine) hamper the growth of mouse tumor xenografts by OXPHOS inhibition sensitive but not resistant cancer cells. What's more, the retrospective study of 62 tumor samples from a clinical trial demonstrates that administration of Berberine reduces the tumor recurrence rate of NNMT^low^/DNMT1^high^ but not NNMT^high^/DNMT1^low^ colorectal adenomas (CRAs). These results thus reveal a critical role of the NNMT‐DNMT1 axis in determining cancer cell reliance on mitochondrial OXPHOS and suggest that NNMT and DNMT1 are faithful biomarkers for OXPHOS‐targeting cancer therapies.

## Graphical abstract

The mitochondrial oxidative phosphorylation (OXPHOS) is a promising target for cancer treatment. NNMT‐DNMT1 axis plays an essential role in maintaining cancer cell sensitivity to OXPHOS inhibition, and the statuses of NNMT and DNMT1 genes are faithful biomarkers for OXPHOS‐targeting cancer therapies.

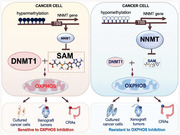

## 1 Introduction

Metabolic reprogramming is a hallmark of cancer cells.^[^ ^1^, ^2^ ^]^ As a central pathway in cellular metabolism, mitochondrial oxidative phosphorylation (OXPHOS) is essential for tumor initiation and progression.^[^ ^1^, ^3^, ^4^, ^5^, ^6^, ^7^ ^]^ There are more than 90 proteins in the five complexes functioning in the OXPHOS pathway.^[^ ^8^ ^]^ The first four complexes (complex I, II, III, and IV) constitute the electron transport chain passing electrons from NADH (oxidized by complex I) or FADH (oxidized by complex II) to oxygen, and energy generated during this process is then used by complex V (also known as ATP synthase) for producing ATP.^[^ ^9^ ^]^ OXPHOS plays important roles in almost all cells; classical OXPHOS inhibitors are often too toxic to be used in clinics for cancer treatment. Recently, a couple of novel OXPHOS inhibitors demonstrating preferential toxicity for cancer cells have been developed and used in preclinical or clinical studies for cancer treatment, exhibiting promising therapeutic efficacy in some tumor models or cancer patients. These OXPHOS inhibitors include: Gboxin, a newly discovered complex V inhibitor exerting toxicity for a significant portion of cancer cell lines;^[^ ^3^ ^]^ IACS010759, a complex I inhibitor conducted in phase I clinic study;^[^ ^10^ ^]^ Berberine, also an OXPHOS complex I inhibitor manifesting protective effect on colorectal carcinogenesis;^[^ ^11^, ^12^ ^]^ and other inhibitors such as Metformin and its analogues, practiced in multiple clinical studies and exhibiting significant antitumor effect.^[^ ^3^, ^10^, ^13^ ^]^ Cultured cancer cells exhibit wide variation in terms of their sensitivities to OXPHOS inhibition.^[^ ^3^, ^14^, ^15^ ^]^ However, the molecular mechanism determining OXPHOS dependency for cancer cells is largely unknown, which curbs the development of cancer therapies targeting this pathway.

NNMT, a metabolic enzyme, catalyzes the reaction that transfers the methyl group from S‐adenosylmethionine (SAM) to nicotinamide, generating S‐adenosylhomocysteine (SAH) and *N*‐methylnicotinamide (MeNAM).^[^ ^16^, ^17^, ^18^ ^]^ MeNAM is further oxidized by aldehyde oxidase and excreted into the urine, which eventually leaves the body.^[^ ^19^ ^]^ Recent works delineate that NNMT is a negative regulator for the intracellular levels of SAM, the universal methyl donor for protein, and DNA methylation.^[^ ^20^, ^21^ ^]^ DNA methylation plays a central role in gene transcription regulation and cell identity determination;^[^ ^22^, ^23^ ^]^ it is tightly regulated by an evolutionally conserved protein family, the DNA methyltransferases (DNMTs).^[^ ^24^ ^]^ Although multiple DNMTs have been identified in the mammalian genome, the genome DNA methylation patterns are mainly established by DNMT3A/DNMT3B and maintained by DNMT1.^[^ ^25^, ^26^ ^]^ Recent work shows that the increased expression of DNMT genes associates with a dynamic turnover of genomic DNA methylation, which plays an essential role in cell lineage determination.^[^ ^23^ ^]^

In this study, through a systematic analysis of gene transcription and DNA methylation of cancer cell lines that are sensitive or resistant to OXPHOS inhibition, we identified that SAM‐regulated enzyme NNMT and SAM consumer DNMT1 function together in maintaining a state of cancer cells for OXPHOS dependency in cultured cancer cells, mouse tumor xenografts, and colorectal adenomas (CRAs) in patients. These data not only reveal an epigenetic mechanism for retrograde regulation of cellular metabolism via modulating mitochondrial function by metabolic enzyme NNMT and epigenetic modifier but also demonstrate that NNMT/DNMT1 might be used as biomarkers for mitochondrial OXPHOS targeting cancer therapies.

## 2 Results

### 2.1 Gboxin Sensitivity Screen Identifies Cancer Cell Lines Sensitive or Resistant to OXPHOS Inhibition

**Figure 1:**
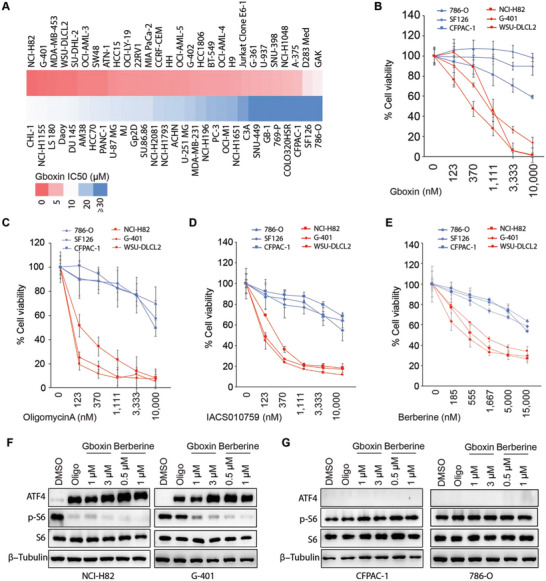
Screen identifies cancer cell lines sensitive or resistant to OXPHOS inhibition. A) Heatmap plot shows results for Gboxin sensitivity screen with a panel of 57 cancer cell lines. After a 3‐day treatment with a series of dilutions of Gboxin, a cell viability assay with CellTilter Glo reagent was performed and Gboxin IC50 was calculated. B) Verification of screen results for Gboxin sensitive (NCI‐H82, G‐401, and WSU‐DLCL2) and resistant (786‐O, SF126, and CFPAC‐1) cancer cell lines. After the cancer cells were incubated with a series dilution of Gboxin for 3 days, cell viability analysis was performed. Mean ± SD. *n* = 3. C–E) Cell viability analysis shows Gboxin sensitive and resistant cancer cell lines are also sensitive or resistant to Oligomycin A, IACS010759, and Berberine, respectively. After cancer cells as indicated were incubated with a series of dilutions of Oligomycin A (C), IACS010759 (D), and Berberine (E) for 3 days, cell viability analysis was performed. Mean ± SD. *n* = 3. F,G) Oligomycin A, Gboxin, and Berberine treatments induce ATF4 while suppressing p‐S6 expression in OXPHOS sensitive (F, NCI‐H82 and G‐401) cancer cells but not in resistant (G, 786‐O and CFPAC‐1) cancer cells. After cancer cells as indicated were treated with Oligomycin A (Oligo, 1 µm), Gboxin (1 and 3 µm) or Berberine (0.5 and 1 µm) for 6 h, western blot assays were applied to detect ATF4 and p‐S6 expression. *n* = 2 at least.

To explore the molecular mechanism underlying cancer cell sensitivity for mitochondrial OXPHOS inhibition, we performed Gboxin (an OXPHOS inhibitor used in preclinical studies) sensitivity screen with a panel of 57 cancer cell lines (**Figure** **1**A). To limit the potential effect of cell growth rate on screen results, we selected cancer cell lines with their cell number doubling time ranging from 19 to 40 h (Table S1, Supporting Information). To exclude potentially biased selection of cancer cells with certain mutations or their original tumor microenvironment, we randomly chose cancer cell lines isolated from tumors origin in a variety of different organs, which included the brain, breast, colon, kidney, blood, liver, lung, lymph, pancreas, prostate, and skin (Table S1, Supporting Information). As a result, this screen identified a significant portion of cancer cell lines that were sensitive to Gboxin with half maximum inhibitory concentration (IC50s) around 1 µm or below; it also identified a portion of cancer cell lines that showed resistance to Gboxin with IC50s greater than 20 µm (Figure 1A). Of note, it seems that Gboxin‐sensitive and resistant cancer cells were neither associated with certain mutations of oncogenes or tumor suppressors nor associated with the organ of origin they isolated from (Table S1, Supporting Information).

Based on the screen results, we selected five Gboxin‐sensitive cancer cell lines (NCI‐H82, G‐401, MDA‐MB‐453, WSU‐DLCL2, and SW48) and four Gboxin‐resistant cancer cell lines (786‐O, CFPAC‐1, GB‐1, and SF126) and performed individual Gboxin sensitivity analysis to check screen quality. As shown in Figure 1B; Figure S1A, Supporting Information, screen results were very reproducible for sensitive cancer cell lines and resistant ones; thus, we picked cell lines (NCI‐H82, G‐401, and WSU‐DLCL2) as representative Gboxin‐sensitive cancer cell lines while cell lines (786‐O, CFPAC‐1, and SF126) were picked as representative Gboxin resistant cancer cells for most of the following studies except that in some cases, data from all nine cell lines were applied as specifically described in the figure legend.

The screen and the corresponding validating experiments were performed with cancer cells cultured in their original medium as recommended by American Type Culture Collection (ATCC) or other source organizations, where these cells were obtained (Table S1, Supporting Information). We further retested Gboxin sensitivity for NCI‐H82, G‐401, WSU‐DLCL2, 786‐O, CFPAC‐1, and SF126 cultured in the same RPMI1640 medium to check whether cancer cell sensitivity to OXPHOS inhibition detected resulted from different culture medium used. As shown in Figure S1B, Supporting information, similar Gboxin sensitivities were observed when the sensitive (NCI‐H82, G‐401, and WSU‐DLCL2) and resistant (786‐O, CFPAC‐1, and SF126) cancer cell lines were growing in the same RPMI1640 medium, implying the existance of an internal mechanism governing OXPHOS dependency for cancer cell viability.

Gboxin primarily targets complex V in the OXPHOS pathway;^[^ ^3^ ^]^ we further checked these Gboxin‐sensitive and resistant cancer cells’ responses to other OXPHOS inhibitors including Rotenone,^[^ ^27^ ^]^ Antimycin A,^[^ ^28^ ^]^ and Oligomycin A^[^ ^29^ ^]^ that target complex I, III, and V, respectively as well as OXPHOS inhibitors IACS010759 and Berberine used in clinical studies for cancer treatment. As shown in Figure 1C–E; Figure S1C,D, Supporting Information, Gboxin‐sensitive cancer cell lines (NCI‐H82, G‐401 and WSU‐DLCL2) were also sensitive to other OXPHOS inhibitors tested, and Gboxin‐resistant cancer cell lines (CFPAC‐1, 786‐O, and SF126) remained resistant to other OXPHOS inhibitors. These data thus imply that Gboxin sensitive and resistant cancer cell lines identified may be sensitive not only to complex V inhibition but also to the inhibition of other OXPHOS complexes, such as complex I and complex III as tested.

OXPHOS dysfunction often leads to upregulation of activating transcription factor (ATF4) and suppression of mammalian target of rapamycin (mTOR) in cancer cells.^[^ ^3^, ^30^, ^31^, ^32^ ^]^ Consistently, we detected a quick and robust activation of ATF4 and suppression of mTOR (reflected by pS6 reduction) upon OXPHOS inhibition in sensitive cancer cells (NCI‐H82, G‐401, and WSU‐DLCL2) when they were treated with Gboxin, Oligomycin A, or Berberine (Figure 1F; Figure S1E, Supporting Information). However, little or no ATF4 activation or mTOR suppression was detected upon OXPHOS inhibition in resistant cancer cells (CFPAC‐1, 786‐O, and SF126) under the same treatments (Figure 1G; Figure S1F, Supporting Information), suggesting very limited induction of cellular stress responses in these cancer cells upon OXPHOS inhibition.

### 2.2 OXPHOS Inhibition Sensitive and Resistant Cancer Cells Have Distinct Cellular Metabolism

**Figure 2:**
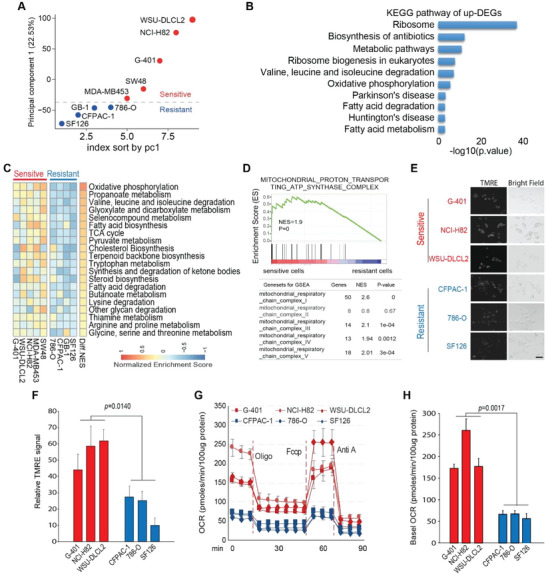
OXPHOS inhibition‐sensitive cancer cells have potentiated mitochondrial metabolism compared with resistant ones. A) Principal component analysis (PCA) with the whole transcriptome of OXPHOS‐sensitive (NCI‐H82, G‐401, MDA‐MB‐453, WSU‐DLCL2, and SW48) and resistant (786‐O, SF126, CFPAC‐1, and GB‐1) cell lines reveal distinct gene transcription pattern between these two groups of cancer cell lines. Red and blue dots represent OXPHOS‐sensitive and resistant cell lines, respectively. B) KEGG enrichment analysis of upregulated genes in OXPHOS‐sensitive cancer cells reveals that five out of the top ten enriched pathways are metabolism related. C) Gene set variation analysis (GSVA) of metabolic‐related pathways shows that genes functioning in mitochondrial OXPHOS, TCA cycle, and pyruvate‐related metabolism pathways are upregulated in OXPHOS‐sensitive cancer cells. Diff NES: difference of normalized enrichment score (sensitive‐resistant). D) Gene set enrichment analysis (GSEA) shows that genes functioning in mitochondrial OXPHOS complexes I, III, IV, and V but not complex II are significantly enriched in OXPHOS inhibition‐sensitive cancer cells compared with those in resistant ones (CFPAC‐1, 786‐O, and SF126). E,F) TMRM staining analysis manifests higher mitochondrial membrane potential in OXPHOS inhibition‐sensitive cancer cell lines (G‐401, NCI‐H82, and WSU‐DLCL‐2) compared with those in resistant ones. E Representative TMRM staining images by fluorescence microscope of indicated cells. Scale bar 20 um. (E). Quantification of (E) by image J software. Mean± SD. *n* = 40. Paired *t*‐test (F). G,H) Basal and maximal oxygen consumption rate (OCR) in OXPHOS inhibition sensitive cancer cells (G‐401, NCI‐H82, and WSU‐DLCL2) is higher than those in resistant ones (786‐O, SF126, and CFPAC‐1). Seahorse analyzer measuring of OCR in OXPHOS inhibition sensitive and resistant cancer cells upon a series treatment as indicated. Cells were treated with Oligomycin A (Oligo,18 min.), Fccp (54 min.), and Antimycin A (Anti A, 72 min.). Mean ± SD; *n* = 3 (G). Quantification of (G) (H). Data was normalized by 100 µg protein. Mean± SD. *n* = 3. Paired *t*‐test.

To explore molecular mechanisms essential for cancer cell sensitivity to OXPHOS inhibition, we performed transcriptome analysis with five OXPHOS sensitive (WSU‐DLCL2, NCI‐H82, G‐401, SW48, and MDA‐MB‐453) and four resistant (GB‐1, 786‐O, CFPAC‐1, and SF126) cancer cell lines. Principal component analysis (PCA) of the whole transcriptome shows that the sensitive and resistant cancer cell lines can be clearly divided into two separated groups (**Figure** **2**A; Figure S2A, Supporting Information) with a significant portion of genes differentially expressed (Figure S2B, Supporting Information). Kyoto Encyclopedia of Genes and Genomes (KEGG) pathway analysis reveals that the enriched pathways of up‐regulated differentially expressed genes (DEGs) in sensitive cancer cells are mostly related to cellular metabolism regulating pathways (Figure 2B), implying that these two groups of cancer cells rely on distinct cellular metabolism. Notably, among all the metabolic related pathways that are differentially expressed, OXPHOS is significantly upregulated in the sensitive cancer cells (Figure 2C; Figure S2C,D, Supporting Information). Besides OXPHOS, other upregulated pathways are those related to propanoate metabolism, branched‐chain essential amino acids degradation, glyoxylate, dicarboxylate metabolism, and so on. (Figure 2C).

OXPHOS consists of five separated complexes (I, II, III, IV, and V) that are located on the mitochondrial inner membrane. Detailed gene set enrichment analysis (GSEA) shows that the genes functioning in complex I, III, IV, and V but not complex II are significantly upregulated in OXPHOS inhibition‐sensitive cancer cell lines compared with the resistant ones (Figure 2D; Figure S2E, Supporting Information). These data were then confirmed by real‐time quantitative PCR (RT‐qPCR, Figure S2F, Supporting Information), implying that there is an enhanced complex I mediated OXPHOS activity in OXPHOS inhibition‐sensitive cancer cells compared with those in the resistant ones. In line with this, OXPHOS inhibition‐sensitive cancer cells display increased mitochondrial membrane potential (Figure 2E,F) and augmented oxygen consumption rate compared with the resistant ones (Figure 2G,H).

The mammalian target of rapamycin (mTOR) pathway is dysregulated in many cancer cells and plays a key role in mitochondrial metabolism regulation. We checked whether the different responses to OXPHOS inhibition are associated with different mTOR activities between cancer cells sensitive or resistant to OXPHOS inhibition. As shown in Figure S2G,H, Supporting Information, no significant difference in baseline mTOR activity was detected between these two groups of cells. Further, no significant change in sensitivity to OXPHOS inhibition was detected after baseline mTOR activity was comprised in both sensitive and resistant cancer cells (Figure S2I–N, Supporting Information). These results thus suggested that cancer cell sensitivity to OXPHOS inhibition might not directly associate with mTOR activity.

**Figure 3:**
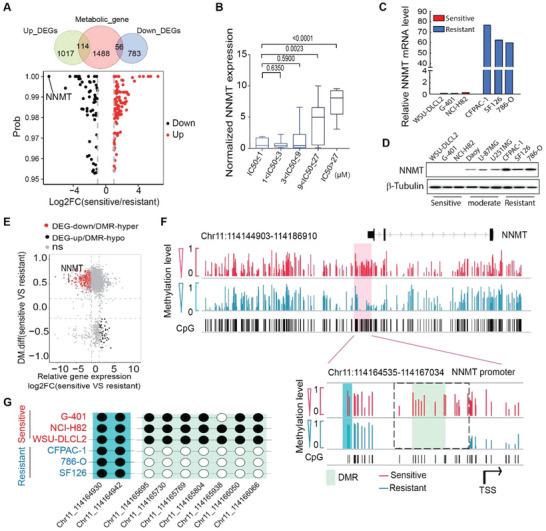
NNMT expression negatively correlated with cancer cell sensitivity to OXPHOS inhibition. A) Venn diagram shows the intersection between the metabolic genes and the differentially expressed genes (DEGs). The volcano plot shows the metabolism‐related DEGs, indicating that NNMT is the most down‐regulated metabolic gene in OXPHOS inhibition‐sensitive cancer cell lines. B) Boxplot shows NNMT transcription negatively correlated with cancer cell sensitivity to Gboxin treatment. 57 cancer cell lines in Gboxin sensitivity screen were divided into five groups according to their Gboxin sensitivity as indicated. NNMT gene transcription data was extracted from DepMap and CCLE and rescaled to a range from 0 to 10. The *p* values by the Mann–Whitney test are shown. C,D) NNMT expression from transcriptome analysis (C) and Western blot (D) with OXPHOS inhibition‐sensitive, moderate sensitive, and resistant cell lines as indicated. E) Scatter plot shows NNMT is one of the most downregulated genes with its promoter DNA methylation increased in OXPHOS sensitive cell lines (NCI‐H82, G‐401, MDA‐MB‐453, WSU‐DLCL2, and SW48) compared with that in resistant ones (786‐O, SF126, CFPAC‐1, and GB‐1). DMR: differentially methylated region. Red dots: down‐regulated DEGs closest to a hyper‐DMR; black dots: up‐regulated DEGs closest to a hypo‐DMR. F) Integrated genome viewer (IGV) shows differential DNA methylation levels on CpG sites in the NNMT promoter region between OXPHOS‐sensitive and resistant cancer cells. Red and blue lines represent the average methylation levels for each CpG site in sensitive and resistant cancer cell lines, respectively. Pale green and aqua backgrounds represent the most differential methylation region and non‐differential methylation region, respectively. The dotted box indicates the DMR. TSS is the abbreviation of the transcription start site. G) DNA methylation at the chosen CpG sites from a non‐DMR (aqua) and the DMR (pale green) in (F), respectively.

Transcriptional analysis reveals that NNMT, a key regulator for the intracellular concentration of SAM,^[^ ^21^, ^33^ ^]^ is the most down‐regulated metabolic gene in OXPHOS‐sensitive cancer cell lines compared with the resistant ones (**Figure** **3**A; Figure S3A, Supporting Information), implying a possible correlation between NNMT expression and cancer cell sensitivity to OXPHOS inhibition. To further probe this possibility, the cancer cell lines tested in our screen were divided into five groups based on their Gboxin sensitivity: group #1 with Gboxin IC50 ≤ 1 µm; group #2 with Gboxin IC50 > 1 µm and ≤3 µm; group #3 with Gboxin IC50 >3 µm and ≤ 9 µm; group #4 with Gboxin IC50 > 9 µm and ≤ 27 µm; and group #5 with Gboxin IC50 > 27 µm. NNMT transcription data for 46 out of these 57 cancer cell lines are available in the public database of Cancer Dependency Map (DepMap) and Cancer Cell Line Encyclopedia (CCLE). As shown in Figure 3B; Figure S3B, Supporting Information, cancer cell sensitivity to OXPHOS inhibition was negatively associated with NNMT transcription level in an exponential manner. Of note, NNMT transcription also showed a strong negative association with the expression of OXPHOS genes across these cancer cell lines (Figure S3C, Supporting Information). We further tested the correlation between NNMT expression and cancer cell sensitivity to OXPHOS inhibition with publicly available data. As shown in Figure S3D–F, Supporting Information, NNMT expression also showed a strong negative association with cancer cell sensitivity to OXPHOS inhibition by small molecular inhibitors (Oligomycin A and Berberine) or OXPHOS gene silencing. The expression of NNMT in OXPHOS sensitive (WSU‐DLCL2, NCI‐H82, and G‐401), moderately sensitive (Daoy, U‐87MG, and U251MG), and resistant (786‐O, CFPAC‐1, and SF126) cancer cells was experimentally verified, which shows undetectable NNMT expression in OXPHOS sensitive cancer cells (Figure 3C,D).

### 2.3 NNMT‐DNMT1 Axis Plays an Essential Role in Maintaining Cancer Cell Sensitivity to OXPHOS Inhibition

An extremely low expression of NNMT in OXPHOS inhibition‐sensitive cancer cells implies this gene might be epigenetically silenced. In fact, the NNMT promoter region is hyper‐methylated in the sensitive cancer cells compared to those in the resistant cells (Figure 3E,F). Consistently, the NNMT expression level is significantly decreased in the sensitive cancer cells in concomitance with the precipitously increased DNA methylation in its promoter region (Figure 3E). Opposite to the negative correlation between NNMT and OXPHOS gene expression, the DNA methylation level in the NNMT promoter positively correlates with OXPHOS gene expression (Figure S3G, Supporting Information). Further analysis identified a differentially methylated region (DMR) within the NNMT promoter, which is hyper‐methylated in the sensitive cancer cells (Figure 3F,G; Figure S3H, Supporting Information). These results thus strongly suggest that DNA methylation plays a critical role in silencing NNMT expression in OXPHOS inhibition‐sensitive cancer cells.

**Figure 4:**
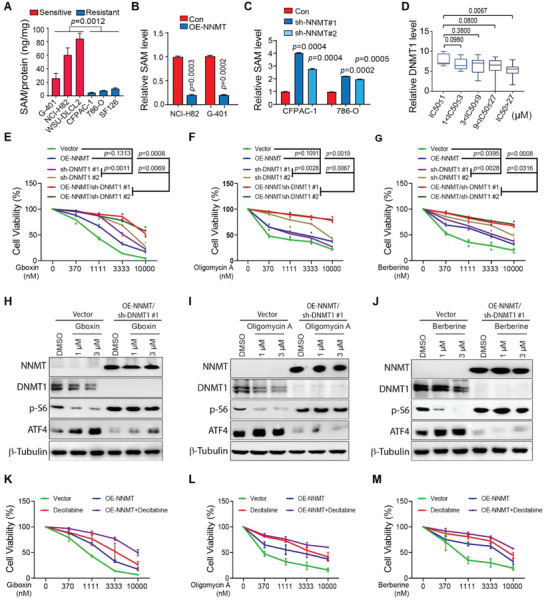
NNMT activation and DNMT1 inhibition render OXPHOS inhibition‐sensitive cancer cells resistant. A–C) NNMT decreases intracellular SAM levels in OXPHOS inhibition‐sensitive and resistant cancer cells. OXPHOS inhibition sensitive cancer cells have higher intracellular SAM levels than that in resistant ones (A); NNMT overexpression decreases intracellular SAM levels in OXPHOS inhibition sensitive cancer cells (NCI‐H82 and G‐401) (B). NNMT knockdown increases intracellular SAM level in OXPHOS inhibition‐resistant cancer cells (CFPAC‐1 and 786‐O); the intracellular SAM level was measured by Elisa analysis and normalized by total protein in indicated cell lines (C). Mean ± SD. *n* = 3. Paired *t*‐test. D) Boxplot shows DNMT1 transcription positively correlated with cancer cell sensitivity to Gboxin treatment. 57 cancer cell lines in Gboxin sensitivity screen were divided into five groups according to their Gboxin sensitivity as indicated. DNMT1 gene transcription data was extracted from DepMap and CCLE, and the expression value of DNMT1 was rescaled to a range from 0 to 10. The *p* value is calculated using the Mann–Whitney test. E–G) NNMT overexpression and DNMT1 knockdown have a synergistic effect on reducing NCI‐H82 sensitivity to treatment of Gboxin (E), Oligomycin A (F), and Berberine (G). NCI‐H82 cells with NNMT overexpression, DNMT1 knockdown, or both were treated with a series dilution of Gboxin, Oligomycin A, and Berberine for 3 days. Cell viability was measured by CellTilter Glo reagent. Two‐way ANOVA test shows the lowest *p* value. *n* = 2. H–J) Western blotting showed attenuated ATF4 upregulation and p‐S6 inhibition in NCI‐H82 cells with NNMT overexpression and DNMT1 knocked down. NCI‐H82 transfected with empty vector or overexpression of NNMT and knockdown of DNMT1 were treated with different concentrations of Gboxin (H), Oligomycin A (I), and Berberine (J) as indicated for 30 h, and ATF4 and p‐S6 expression were measured by Western blot. *n* = 2 at least. K–M) NCI‐H82 cells with NNMT overexpression, Decitabine treatment or both were incubated with a series dilution of Gboxin (K), Oligomycin A (L), and Berberine (M) for 3 days. Cell viability was then measured by CellTilter Glo reagent. Paired *t*‐test shows the lowest *p* value compared to the vector. Mean ± SD. *n* = 2.

NNMT has been identified as a negative regulator for the intracellular level of SAM,^[^ ^21^, ^33^, ^34^ ^]^ which is converted to SAH in the methionine cycle and thus the intracellular level of SAM and SAH are often inversely correlated. Consistent with lower NNMT expression in cancer cells sensitive to OXPHOS inhibition, the intracellular level of SAM in these cancer cells is much higher than that in the resistant ones (**Figure** **4**A). Importantly, overexpression of NNMT reduces SAM and global methylation levels but increases SAH concentration in cancer cells sensitive to OXPHOS inhibition (Figure 4B; Figure S3I–L, Supporting Information), while knockdown of NNMT in cancer cells resistant to OXPHOS inhibition increases SAM (Figure S3M, Supporting Information; Figure 4C). Taken together, these data thus suggest that low NNMT expression leads to increased SAM levels in OXPHOS inhibition‐sensitive cancer cells compared with that in resistant cancer cells.

SAM is the universal methyl donor for protein and DNA methylation. Interestingly, molecular function enrichment analysis of the up‐regulated DEGs in OXPHOS inhibition‐sensitive cancer cells identifies DNA binding and methyltransferase activities (Figure S4A, Supporting Information). DNA methylation is established by two methyltransferases, DNMT3A and DNMT3B, and maintained by another DNA methyltransferase DNMT1 in a complex with UHRF1.^[^ ^35^ ^]^ As shown in Figure S4B–D, Supporting Information, the expression of DNMT1, UHRF1, and DNMT3A is inversely associated with the expression of NNMT in cancer cell lines as well as in multiple types of tumors, such as glioblastoma multiforme (GBM), rectum adenocarcinoma (READ), breast cancer (BRCA), and lung squamous cell carcinomas (LUSC) as analyzed. Although it seems that expressions of DNMT1, UHRF1, and DNMT3A are also upregulated in OXPHOS inhibition‐sensitive cancer cells identified in our screen, only DNMT1 expression exhibits a significantly positive correlation with cancer cell sensitivity to OXPHOS inhibition (Figure 4D; Figures S4E–H and Figure S5A, Supporting Information). A positive correlation between DNMT1 expression and cancer cell sensitivity to OXPHOS inhibition was also detected with publicly available data from Depmap (Figure S4I–K, Supporting Information). Taken together, these data thus imply an enhanced DNA methylation activity in OXPHOS sensitive cancer cells compared with those in the resistant ones. Although no significant difference in DNA methylation at OXPHOS gene promoters was detected between OXPHOS‐sensitive and resistant cells, global DNA methylation levels in OXPHOS‐sensitive cancer cells are higher compared with those in the resistant ones (Figure S5B,C, Supporting Information).

We then postulated that it might be the NNMT/DNMTs axis‐mediated DNA methylation that plays an important role in cancer cell sensitivity to OXPHOS inhibition. Although DNMT3A and DNMT3B are essential for de novo DNA methylation, DNMT1 is the central enzyme required for the maintenance of DNA methylation through cell proliferation. To check whether DNA methylation governed by DNMTs plays a role in the regulation of OXPHOS dependency for cancer cells, we examined cancer cell sensitivity to OXPHOS inhibition after manipulating the expressions of DNMT1 or NNMT or both. As shown in Figure 4E–G; Figure S5D, Supporting Information, NNMT overexpression or DNMT1 knockdown itself moderately reduced NCI‐H82 sensitivity to the OXPHOS inhibitors (Gboxin, Oligomycin A, and Berberine), and NNMT overexpression and DNMT1 knockdown at the same time exhibited an additive effect on decreasing NCI‐H82 sensitivities to these OXPHOS inhibitors. The reduced sensitivity to OXPHOS inhibition of cancer cells upon NNMT overexpression and DNMT1 knockdown was also reflected by attenuated ATF4 upregulation and p‐S6 downregulation when treated with OXPHOS inhibitors (Gboxin, Oligomycin A, and Berberine) (Figure 4H–J). In line with these findings, NNMT overexpression and DNMT1 knockdown reduced OXPHOS gene expression, mitochondrial membrane potential, and the OCR in cancer cells sensitive to OXPHOS inhibition (Figure S5E–G, Supporting Information). Interestingly, treatment of these OXPHOS inhibitors (Gboxin, Oligomycin A, and Berberine) decreases DNMT1 expression in OXPHOS inhibition‐sensitive cancer cells (Figure 4H–J), implying a reciprocal regulation between mitochondrial metabolism and epigenetic regulation.

Decitabine, a DNMT1 inhibitor, depletes DNMT1 through proteasomal degradation.^[^ ^36^ ^]^ Similar to DNMT1 knockdown, Decitabine treatment itself and together with NNMT overexpression has an additive effect on reducing cancer cell (NCI‐H82 and G‐401) sensitivities to OXPHOS inhibitors, Gboxin, Oligomycin A, and Berberine, (Figure 4K–M; Figure S5H–J, Supporting Information). All these data thus suggest that SAM‐regulating protein NNMT and SAM‐consuming enzymes DNMTs function together in maintaining a cancer cell state, which is sensitive to OXPHOS inhibition. However, NNMT inhibition and DNMT1 overexpression are not able to render OXPHOS‐resistant cancer cells (786‐O and CFPAC‐1) sensitive (data not shown), likely because enhanced DNMT1 activity itself is not able to create a genome‐wide DNA methylation status sufficient for altering cancer cells’ sensitivity to OXPHOS inhibition.

### 2.4 NNMT^low^/DNMT1^high^ Defines OXPHOS Inhibition‐Sensitive Tumors Both in Mouse Tumor Xenografts and in Patients with CRAs

**Figure 5:**
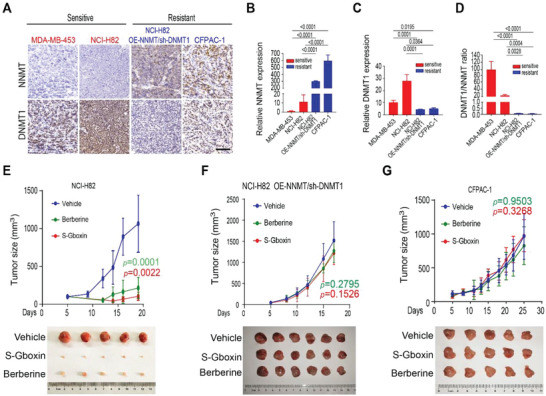
OXPHOS inhibitors, S‐Gboxin and Berberine, inhibit growth of xenograft tumors by OXPHOS sensitive cells but not resistant ones. A–D) OXPHOS inhibition‐sensitive and resistant cancer cells maintain their NNMT and DNMT1 status in vivo. 5 × 10^6^ NCI‐H82 (sensitive to OXPHOS inhibition), MDA‐MB‐453 (sensitive to OXPHOS inhibition), NCI‐H82‐OE‐NNMT/sh‐DNMT1 (resistant to OXPHOS inhibition), and CFPAC‐1 (resistant to OXPHOS inhibition) cancer cells were subcutaneously injected into flanks of nude mice. Mice were sacrificed when tumor volume (1/2 × length × width^2^) was ≈1000 mm^3^. Representative immunohistochemistry staining images show expression of NNMT and DNMT1 in tumor samples by cancer cells as indicated. Scale bar: 100 um (A). Quantification of NNMT expression (B), DNMT1 expression (C), and DNMT1/NNMT ratio (D) of (A). Mean± SD. *n* = 3. One‐way ANOVA test. E–G) NCI‐H82 (E), NCI‐H82‐OE‐NNMT/sh‐DNMT1 (F), and CFPAC‐1 (G) cancer cells were subcutaneously injected into flanks of nude mice as in (A). 5 days after transplant, mice were administrated intraperitoneally with S‐Gboxin (10 mg kg^−1^ per day) or Berberine (10 mg kg−^1^ per day). Up panel, growth for NCI‐H82 (E), NCI‐H82‐OE‐NNMT/sh‐DNMT1 (F), and CFPAC‐1 (G) tumors were assessed every 2 to 3 days and calculated with the formula (1/2 × length × width^2^). Tumor *n* = 7 for NCI‐H82; *n* = 9 for NCI‐H82‐OE‐NNMT/sh‐DNMT1; and *n* = 9 for CFPAC‐1 xenografts. Paired *t*‐test shows the lowest *p* value compared to vehicle. Bottom panel, images show five or six NCI‐H82 (E), NCI‐H82‐OE‐NNMT/sh‐DNMT1 (F), and CFPAC‐1 (G) tumors each on the day all mice were sacrificed after the treatments as indicated.

We then check whether the cancer cell's sensitivity to OXPHOS inhibition observed in vitro is conserved in vivo. To test this, OXPHOS inhibition‐sensitive (NCI‐H82 and MDA‐MB‐453) and resistant (CFPAC‐1 and NCI‐H82‐OE‐NNMT/sh‐DNMT1) cancer cells are subcutaneously injected into flanks of nude mice. As shown in **Figure** **5**A–D, expression of NNMT remains low in tumor xenografts driven by OXPHOS inhibition‐sensitive cancer cell lines (NCI‐H82 and MDA‐MB‐453) compared with those driven by the resistant cancer cells (CFPAC‐1 and NCI‐H82‐OE‐NNMT/sh‐DNMT1), while the opposite expression pattern is observed for DNMT1 (Figure 5A–D). These data thus suggest that the status of the NNMT/DNMT1 axis detected in vitro for OXPHOS sensitive and resistant cancer cells remains when these cells are growing in vivo.

We then treated these tumor‐bearing mice by intraperitoneal (i.p.) injection of S‐Gboxin (a stable version of Gboxin) or Berberine, both of which have suitable plasma stability and tumor penetration^[^ ^3^ ^]^ (Figure S6A,B, Supporting Information). As shown in Figure 5E; Figure S6C, Supporting Information, treatments of S‐Gboxin and Berberine inhibit tumor cell proliferation and growth of xenograft tumors driven by OXPHOS inhibition sensitive cancer cells (NCI‐H82 and MDA‐MB‐453) without inducing obvious toxicity (Figure S6D, Supporting Information). Similar to what was observed in vitro, treatments of S‐Gboxin and Berberine lead to reduced DNMT1 expression in these tumors (Figure S6E–H, Supporting Information). DNMT1 inhibition and NNMT activation render OXPHOS‐sensitive cancer cells resistant in vitro (Figure 4E–M); we thus further examined the effects of S‐Gboxin and Berberine on tumor xenografts by NCI‐H82 cancer cells with NNMT overexpression and DNMT1 knockdown (NCI‐H82‐OE‐NNMT/sh‐DNMT1). NCI‐H82‐OE‐NNMT/sh‐DNMT1 tumors remain similar in growth rate in vivo to their parental NCI‐H82 tumors (Figure 5F; Figure S6I,J, Supporting Information). However, opposite to the greatly reduced growth of NCI‐H82 tumors, treatments of S‐Gboxin and Berberine have no significant effect on growth of NCI‐H82‐OE‐NNMT/sh‐DNMT1 xenografts. Similarly, S‐Gboxin and Berberine treatments also exhibit no inhibitory effects on tumors driven by CFPAC‐1, an OXPHOS‐resistant cancer cell line (Figure 5G; Figure S6K,L, Supporting Information). These data thus strongly suggest that OXPHOS sensitive and resistant cancer cells identified in vitro maintain their NNMT/DNMT1 expression features and sensitivity to OXPHOS inhibition in vivo.

**Figure 6:**
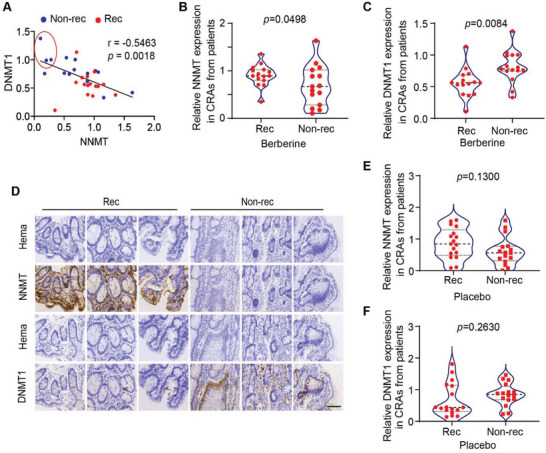
NNMT^low^/DNMT1^high^ expressing colorectal adenomas (CRAs) are Berberine responders. A–C) 30 CRA samples from patients with non‐recurrent (15 samples) or recurrent (15 samples) tumors receiving Berberine treatment in the clinical trial as described were randomly selected. All CRA samples were immunohistochemically stained for detecting NNMT and DNMT1 expression. NNMT and DNMT1 expression were then quantified. Scatter plot shows the negative correlation between NNMT and DNMT1 expression in the 30 CRAs. Blue dots: CRAs from patients with nonrecurrent tumors; red dots: CRAs from patients with recurrent tumors. Three CRAs in the red circle highlight those patients with NNMT^low^/DNMT1^high^ CRAs who did not have recurrent tumors after Berberine treatment. The correlation value was calculated by the Pearson correlation coefficient method and the *p* value was calculated by two‐tailed Pearson's Correlation (A). Violin plot shows a significantly lower expression of NNMT in CRAs from the patients with non‐recurrent tumors compared to those from the patients with recurrent tumors after Berberine treatment. The *p* value was calculated by paired *t*‐test (B). Violin plot shows significantly higher expression of DNMT1 in CRAs from patients with non‐recurrent tumors compared with that in patients with recurrent tumors after Berberine treatment. *P* value was calculated by paired *t*‐test (C). D) Representative immunohistochemical images showed expression of NNMT and DNMT1 in three CRAs from patients with recurrent tumors (left panel) and in three CRAs from patients with non‐recurrent tumors as in red circle in (A) (right panel). All CRAs analyzed were from Berberine‐treated participants. Scale bar 100 µm. E,F) 16 CRA samples from patients with non‐recurrent or recurrent tumors after placebo treatment in the clinical trial as described were randomly selected. All tumor samples were immuno‐stained for detecting NNMT and DNMT1 expression. NNMT and DNMT1 expression were then quantified and presented. The violin plot showed no significant difference in NNMT (E) and DNMT1 (F) expression in CRAs from the patients with non‐recurrent and recurrent tumors after placebo treatment. The *p* value was calculated by paired *t‐*test.

Recently, a large clinical study with 891 participants for evaluating Berberine's efficacy in the prevention of recurrence of colorectal adenoma (CRA) was carried out.^[^ ^12^ ^]^ Berberine treatment effectively reduced CRA recurrence rate by ≈11% (recurrence rate is 47% in placebo‐treated group while 36% in Berberine treated group) implying a heterogenous response of CRAs to Berberine treatment.^[^ ^12^ ^]^ In order to assess the possible correlation between Berberine responders and the status of NNMT/DNMT1, we did a retrospective study with the CRA samples collected in this clinical trial. 15 recurrent CRA samples and 15 nonrecurrent CRA samples in Berberine treated group were available and selected to perform the immunology staining for analyzing NNMT and DNMT1 expression. As shown in **Figure** **6**A, expression of NNMT showed a strong negative correlation with expression of DNMT1 among the total 30 recurrent/nonrecurrent CRAs from the Berberine treated group. Consistent with our findings in cultured cancer cell lines (Figure 1) and tumor models (Figure 5) that NNMT^low^/DNMT1^high^ cancer cells are sensitive to OXPHOS inhibition, nonrecurrent CRAs (enriched with Berberine sensitive tumors) had significant lower NNMT but higher DNMT1 expression compared with recurrent CRAs (Berberine resistant ones) (Figure 6B,C). Of note, three CRAs with the highest DNMT1 but lowest NNMT expression were all nonrecurrent tumors in Berberine treated group (Figure 6A,D). Importantly, pathology analysis of these three NNMT^low^/DNMT1^high^ CRAs revealed that two of them were advanced adenomas and likely recurrent shortly after complete tumor resection implying that these CRAs were Berberine responders (data not shown). To further exclude the possibility that NNMT^low^/DNMT1^high^ labels a group of less aggressive CRAs that are native nonrecurrent tumors, we randomly selected 16 recurrent and 16 nonrecurrent CRA samples in placebo‐treated group and performed the same immunostaining for analyzing NNMT and DNMT1 expression. As shown in Figure S6M, Supporting Information, although NNMT and DNMT1 also exhibit a strong negative correlation across all 32 CRA samples, neither NNMT nor DNMT1 show a significant expression difference between recurrent CRAs and non‐recurrent CRAs in the placebo‐treated group (Figure 6E,F) suggesting no significant correlation between the status of NNMT/DNMT1 and CRA recurrence in untreated patients. Taking together, all these data strongly suggest that it is the NNMT^low^/DNMT1^high^ CRAs that are sensitive to Berberine treatment.

## 3 Discussion

**Figure 7:**
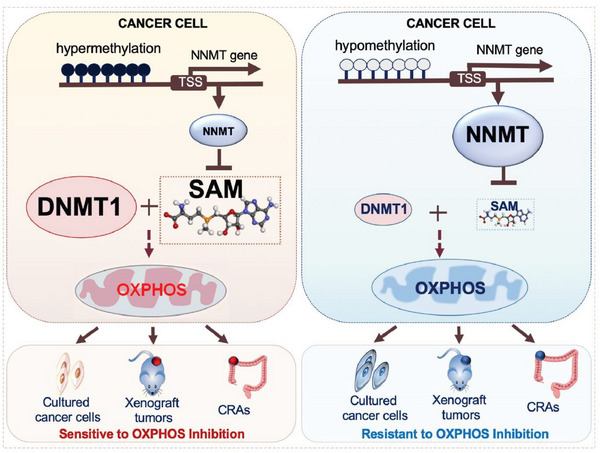
Model for the role of NNMT‐DNMT1 axis in maintaining cancer cell sensitivity to OXPHOS inhibition. Low expression of NNMT1, a negative regulator for intracellular SAM level, and high expression of DNMT1, a SAM‐consuming enzyme, play an essential role in maintaining a cancer cell state that is sensitive to OXPHOS inhibition. This mechanism is conserved in cultured cancer cells, mouse tumor xenografts, and human CRAs.

In this study, through a screen of cancer cell lines isolated from tumors of multiple organs of origin, we found cancer cells vary greatly in terms of their sensitivities to OXPHOS inhibitors used in clinical or preclinical studies. After a systematic analysis of OXPHOS sensitive and resistant cancer cell lines, we revealed that SAM‐regulated enzyme NNMT and SAM‐consuming DNA methyltransferase DNMT1 negatively and positively correlated with cancer cell sensitivity to OXPHOS inhibition, respectively. NNMT overexpression and DNMT1 downregulation exhibit additive effects on reducing cancer cell sensitivity to OXPHOS inhibition, suggesting these proteins function together in maintaining a cancer cell state, which is sensitive to OXPHOS inhibition. However, our results also show that NNMT inhibition and DNMT1 overexpression cannot render OXPHOS‐resistant cancer cells sensitive. This is likely because DNMT1 is not a de novo DNA methyl transferase and it cannot generate a DNA methyl pattern critical for cancer cell dependency of OXPHOS by itself. Nevertheless, importantly our data from mouse tumor models and the retrospective study of samples from a clinical trial aiming for checking the role of Berberine on tumor recurrence reveals that the regulation for mitochondrial OXPHOS reliance of cancer cells by NNMT^low^/DNMT1^high^ is conserved among cancer cell lines, mouse tumor models, and tumor patients (**Figure** **7**).

Both metabolic reprogramming and epigenetic remodeling are established cancer hallmarks, and they reciprocally regulate each other.^[^ ^37^ ^]^ It is now well established that mutations in certain mitochondrial enzymes alter epigenetic modification and promote tumorigenesis; these enzymes include isocitrate dehydrogenase 1/2 (IDH1/2), succinate dehydrogenase complex flavoprotein subunit A (SDHA), and fumarate hydratase (FH).^[^ ^38^, ^39^ ^]^ However, known key players in epigenetic modification regulating mitochondrial metabolism are not well explored. In this work, we found that NNMT^low^/DNMT1^high^ expressing cancer cells possess increased OXPHOS gene expression, upregulated mitochondrial metabolism, and potentiated sensitivity to OXPHOS inhibition. Although the increased DNMTs and SAM in OXPHOS inhibition‐sensitive cancer cells may result in augmented global DNA methylation, no significant alternation of methylation level in promoter regions of the upregulated OXPHOS genes was detected, suggesting the increased expression of OXPHOS genes is unlikely the direct target for DNMTs; and thus, the direct NNMT/DNMT target(s) essential for cancer cell sensitivity to OXPHOS inhibition remain largely unknown and need further exploration. However, merging reports show increased expression of DNMTs often associate with a more dynamic turnover of DNA methylation that is critical for cell lineage determination.^[^ ^40^, ^41^, ^42^, ^43^ ^]^ Thus, the increased expression of DNMTs in OXPHOS‐sensitive cancer cells detected in this study might imply that these cancer cells resemble certain cell lineage status at which mitochondrial energetics is essential for their viabilities.

Recently, emerging mitochondrial OXPHOS inhibitors have been developed or discovered and used in preclinical or clinical studies.^[^ ^14^, ^44^ ^]^ Although certain tumor type seems more sensitive to OXPHOS inhibition,^[^ ^45^ ^]^ the precise mechanism for cancer OXPHOS dependency remains elusive and excellent biomarkers are still lacking, which greatly limits the development of novel cancer therapies by applying mitochondrial OXPHOS inhibitors. In this study, we found expression of NNMT negatively associates with OXPHOS gene expression and cancer cell sensitivity to OXPHOS inhibition. NNMT expression is extremely low in OXPHOS‐sensitive cancer cells with its gene promoter region highly methylated. DNMT1 expression goes on contrary to NNMT. Thus, the status of NNMT and DNMT1 in cancer cells provides an excellent base for developing biomarkers for selecting cancer patients suitable for therapies targeting the mitochondrial OXPHOS pathway. Indeed, our data with CRA samples from an 891‐patient clinical trial demonstrates a promising prediction of CRA patients responding to the reagent of Berberine, which inhibits OXPHOS in cancer cells.

## 4 Experimental Section

### Patient Specimens

Paraffin‐embedded patient samples were obtained from the Western Campus of Renji Hospital, Shanghai Jiao Tong University School of Medicine. This study was approved by the ethics committee of Renji Hospital (RA‐2019‐349), Shanghai Jiao Tong University School of Medicine, and the enrolled subject signed the informed consent form.

### Cell Culture

NCI‐H82, G‐401, MDA‐MB‐453, SW48, 786‐O, CFPAC‐1 cells, and HEK293T were sourced from ATCC, WSU‐DLCL2 cell was from Deutsche Sammlung von Mikroorganismen und Zellkulturen (DSMZ) and SF126 cell was from JCRB Cell Bank. NCI‐H82, G‐401, WSU‐DLCL2, and 786‐O were cultured in 1640 medium (WISENT, 350‐000‐CL) with 10% FBS, 1% Pen/Strep. SW48, SF‐126, and HEK293T were cultured in DMEM medium (WISENT, 319‐005‐CL) with 10% FBS, 1% Pen/Strep. CFPAC‐1 were cultured in IMDM medium (MINGZHOUBIO, MPM150510) with 10% FBS, 1% Pen/Strep. MDA‐MB‐453 was cultured in L‐15 medium (WISENT, 323‐050‐CL) with 10% FBS, 1% Pen/Strep. MDA‐MB‐453 was maintained in 100% air at 37 °C and other cells were in 5% CO_2_ at 37 °C.

### Cell Viability Assay

Cells (0.5–1 × 10^3^ cells per well) were seeded in 96‐well plate and incubated with or without indicated reagents for 72 h or as specified. Cell viability was determined by CellTilter Glo Luminescent Cell Viability Assay (Promega, G7572).

### Generation of Gene Overexpression and Knockdown Cell Line

The full length of cDNA encoding human NNMT was generated from 786‐O cell mRNA and subcloned into pLVX‐Puro vector. DNMT1 gene knockdown shRNA sequence was subcloned into pLVX‐shRNA2 Vector. These vectors were transfected into HEK29T cell with packaging plasmids psPAX2 and PMD.2G at a ratio of 6:2:1 to get specific lentiviruses. Target cells, NCI‐H82, G401, WSU‐DLCL2, 786‐O, and CFPAC‐1, were then infected by lentiviruses and selected using puromycin or fluorescence. Sequences used for DNMT1 targeting shRNAs were:

#1: CCCAATGAGACTGACATCAA

#2: CCGAATACATTCTGATGGAT

### Preparation of RNA‐Seq Library

For RNA‐seq, 1 × 10^6^ cells were collected for each sample. Briefly, total RNA was extracted using TRIZOL Reagent (Life Technologies, 15596‐018) according to the manufacturer's instructions. RNA samples with an RNA integrity number (RIN) of more than 7.0 were used for library preparation. After ribosomal RNA depletion, fragmented, first, and second strand cDNA were synthesized, dA‐tailed, and adapter‐ligated using TruSeq Stranded Total RNA with Ribo‐Zero Gold kit (Illumina, 20020598); the libraries were generated by PCR. Following quantification by Agilent Bioanalyzer 2200, 150 bp paired‐end sequencing was performed on an Illumina Novaseq 6000 sequencer.

### DNA Extraction and Bisulfite Genomic Sequencing PCR (BSP)

DNA was purified using TIANamp Genomic DNA Kit (TIANGEN, DP304). Then, the DNA was treated with bisulfite and purified using EZ DNA METHYLATION‐GOLD (Zymo, D5006). For PCR, primers were designed by bisearch.^[^ ^46^ ^]^ The PCR product was analyzed by Sanger sequencing. The following primers were used.

Forward #1: AAGGGTTATAATAAAAGAGAGAG

Reverse #1: CCTTTCACTTAATTCCAAAAA

Forward #2: TGGAATTAAGTGAAAGGATGATT

Reverse #2: AAACAAAAAAATAACATAAACCC

Forward #3: TGGGTATAAAAAATAGTTAGAGG

Reverse #3: CCAAACAACTAAAACTACAAA

### SAM and SAH Assay

SAM (Shanghai Jianglai, JL48140) and SAH (XinYu Biology, XYH9132681) were measured using an enzyme‐linked immunosorbent assay (ELISA) kit according to the manufacturer's instructions. In brief, cells were lysed with lysis buffer (Keygentec, KGP1100). The supernatant was collected after centrifugation for 10 min at 12 000 *×* g. Metabolites in the supernatant were captured in the 96‐well microplate. HRP conjugated secondary antibody was added and bonded to the detection antibody. After adding the substrate, the data were read and analyzed via the absorbance of 450 nm in a microplate reader.

### Western Blots and Antibodies

Cells were washed in cold PBS and lysed in protein lysis buffer (Keygentec, KGP1100) for 0.5 h at 4 °C. The supernatant was collected after centrifuging for 10 min at 12 000 × *g*. Protein concentration was measured by the BCA Protein Assay kit (Keygentec, KGP903). Proteins were separated on ≈10–12% SDS‐PAGE gels. Following incubation with primary antibody and HRP conjugated secondary antibodies, signals were captured using ChemiScope 6200 Chemiluminescence imaging system (CLINX). The following antibodies were used, rabbit anti‐ATF4 (1:500, Santa, SC‐200), rabbit anti‐p‐S6 (1:1000, CST, 4856S), rabbit anti‐S6 (1:1000, CST, 2217S), rabbit anti‐*β*‐actin (1:1000, Proteintech, 20536‐1‐AP), rabbit anti‐*β*‐tubulin (1:1000, CST, 2146S), rabbit anti‐NNMT (1:1000, Proteintech, 15123‐1‐AP), and rabbit anti‐DNMT1 (1:1000, CST, 5032).

### RNA Isolation and qPCR

Total RNA was extracted by RNA isolater Total RNA Extraction Reagent (Vazyme, R401‐01) according to the manufacturer's instructions. cDNA was synthesized by PrimeScript RT reagent Kit with gDNA Eraser kit (Takara, RR047A). qPCR was performed using ChamQ Universal SYBR qPCR Master Mix (Vazyme, Q711‐02). The primers used were listed below.

MTCO1 – Forward Primer: GACGTAGACACACGAGCATATTTCA

MTCO1 – Reverse Primer: AGGACATAGTGGAAGTGAGCTACAAC

MTND4 – Forward Primer: AAGTCAAAAAGCTATTA

MTND4 – Reverse Primer: CTTACATCCTCATTACTATTC

MTND4L – Forward Primer: TCGCTCACACCTCATATCCTC

MTND4L – Reverse Primer: AGGCGGCAAAGACTAGTATGG

NDUFA3 – Forward Primer: GGTCGTGTCCTTCGTCGTC

NDUFA3 – Reverse Primer: ACCTCTGATCACACACATGC

MTND2 – Forward Primer: AGCACCACGACCCTACTACT

MTND2 – Reverse Primer: TGGTGGGGATGATGAGGCTA

ATP6V0A1‐ Forward Primer: AGGCTGAAATCGAGAACCCC

ATP6V0A1‐ Reverse Primer: GCTCGGAACCCTTCACAGAT

MTCO3 – Forward Primer: TCACCCCGCTAAATCCCCTA

MTCO3 – Reverse Primer: TGACGTGAAGTCCGTGGAAG

MTND3 – Forward Primer: GCGGCTTCGACCCTATATCC

MTND3 – Reverse Primer: AGGGCTCATGGTAGGGGTAA

MTCYB – Forward Primer: ATCACTCGAGACGTAAATTATGGCT

MTCYB – Reverse Primer: TGAACTAGGTCTGTCCCAATGTATG

MTATP6 – Forward Primer: TAGCCATACACAACACTAAAGGACGA

MTATP6 – Reverse Primer: GGGCATTTTTAATCTTAGAGCGAAA

MTND1‐Forward Primer: CCTCCTACTCCTCATTG

MTND1‐Reverse Primer: TAGATGTGGCGGGTT

SDHC – Forward Primer: CTGTTGCTGAGACACGTTGGT

SDHC – Reverse Primer: ACAGAGGACGGTTTGAACCTA

MTCO2 – Forward Primer: GACCTGCGACTCCTT

MTCO2 – Reverse Primer: CGTGTAGCGGTGAAA

THND5 – Forward Primer: TACTCGGATTCTACCCT

THND5 – Reverse Primer: GTGGAGATTTGGTGCT

THATP8 – Forward Primer: ACAAACTACCACCTACC

THATP8 – Reverse Primer: AATGAAGCGAACAGAT

UQCRC 2‐ Forward Primer: TTCAGCAATTTAGGAACCACCC

UQCRC2 – Reverse Primer: GGTCACACTTAATTTGCCACCAA

SDHA – Forward Primer: CAAACAGGAACCCGAGGTTTT

SDHA – Reverse Primer: CAGCTTGGTAACACATGCTGTAT

SDHB – Forward Primer: ACAGCTCCCCGTATCAAGAAA

SDHB – Reverse Primer: GCATGATCTTCGGAAGGTCAA

NNMT – Forward Primer: GAGATCGTCGTCACTGACTACT

NNMT – Reverse Primer: CACACACATAGGTCACCACTG

Actin – Forward Primer: CATGTACGTTGCTATCCAGGC

Actin – Reverse Primer: CTCCTTAATGTCACGCACGAT

### Mitochondrial Membrane Potential (MMP) Measurement

MMP measurement was performed with TMRM mitochondrial membrane potential assay kit (Invitrogen, T668, Tetramethylrhodamine, Catalog Number I34361). In brief, cells were incubated with TMRM (200 nm) for 30 min at 37 °C, then samples were analyzed by flow cytometry (CytoFLEX LX, BECKMAN COULTER) or captured by Olympus microscopy.

### OCR Measurement

OCR was determined by Seahorse Flux Analyzer XF96 (Agilent) according to the manufacturer's instructions. In brief, 80% of confluent cells were equilibrated for 1 h at 37 °C without CO_2_. OCR was then measured before and after sequential injection of indicated compounds in the corresponding figure.

OCR observation was also determined by Agilent MitoXpress Xtra (XF200 Oxygen Consumption Assay). In brief, NCI‐H82 cells were seeded at a concentration of 80 000/90uL/well in a 96‐well plate. Then, there was addition with 10 uL Mitoexpress Xtra reagent following covered with 100 uL mineral oil. Read the plate immediately at 37 °C in a fluorescence plate reader (380 nm for excitation and 650 nm for emission) for 2 h and then collect the data.

### Xenograft Mouse Model

All mouse experiments were performed in accordance with protocols approved by the Institutional Animal Care and Use Committees (IACUC) at Tongji University with approved ID number SHDSYY‐2022‐P0032. 5‐weeks old BALB/c nude female mice were bought from Shanghai SLAC Laboratory Animal Company. 5 × 10^6^ cells were then subcutaneously injected into the flanks of the nude mouse at a volume of 100 µL. After 5 days, mice were treated with S‐Gboxin (10 mg kg^−1^ day) or Berberine (10 mg kg^−1^ per day) by intraperitoneal injection. Tumor volume was calculated using the following formula, length × width^2^ × 0.5.

### Immunohistochemistry and Calculation

Tumors were fixed in 4% paraformaldehyde overnight and then paraffined embedded. After dewaxing, paraffin‐embedded sections were rehydrated in gradient diluted alcohol. Further antigen retrieval was carried out before primary antibody incubation overnight at 4 °C. After washing, a secondary antibody was added to the slide and incubated for 1 h at 37 °C. Slides were then stained with hematoxylin and sealed. Images were captured by Olympus microscopy. The expression value of indicated protein was measured by the integrated density of indicated protein divided by that of the nucleus in Image J software.

The following antibodies were used, mouse anti‐DNMT1 (1:50, Santa, A1020), rabbit anti‐NNMT (1:50, Proteintech, 15123‐1‐AP), and rabbit anti‐Ki67 (1:200, CST, 12202).

### Immunofluorescence Staining for Global Methylation Analysis

Cells were cultured on poly‐lysine coated dish overnight. Immunofluorescence staining was then performed according to a previous study.^[^ ^47^ ^]^ In brief, permeabilized cells were processed in blocking solution (0.1% Triton X‐100 and 5% BSA in PBS) for 1 h at room temperature. Then, the samples were treated with 1.5 m HCl for 1 h. After washing with PBS, cells were incubated with 5‐methylcytosine primary antibodies (1:500, proteintech, 61480) at 4 °C overnight. Subsequently, the samples were incubated with the fluorescence tagged secondary antibody (1:500, proteintech, SA00013‐1) for 1 h at room temperature. Following staining with Hoechst 33342, images were captured by Olympus microscopy.

### RNA‐Seq Data Processing and Analysis

First, cutadapt (V1.18) was used to remove adaptor sequences, low‐quality bases, and reads shorter than 25 bases with parameters “‐a AGATCGGAAGAGC ‐A AGATCGGAAGAGC –trim‐n ‐m 25 ‐q 20,20”. Next, the trimmed clean data were mapped to hg19 reference genome using Hisat2 (V2.1.0) with parameters “–dta‐cufflinks –no‐discordant –rna‐strandness RF”. After that, gene expression levels were quantified as Fragments Per Kilobase per Million mapped reads (FPKM) by stringtie (V1.3.4d). Genes with FPKM < 1 in all samples were considered no expression.

Gene expression comparisons between OXPHOS inhibition sensitive and resistant cancer cells were performed by NOISeq (V2.30.0) with parameters “cv.cutoff = 100”. Then, the differentially expressed genes (DEGs) were identified with a probability (prob) more than 0.95, fold change (FC) of FPKM larger than 2 or 1.5, and the max FPKM in two groups greater than 3.

### Functional Annotations and Enrichment Analysis

To annotate the enriched functions of DEGs, Gene Ontology (GO) enrichment analyses of molecular function and KEGG pathway annotation in DAVID Bioinformatics Resources (v6.8) were performed with default settings.

The Gene Set Enrichment Analysis (GSEA, V3.0) was also performed, in which the list of genes was ranked by the expression fold change of the OXPHOS inhibition sensitive/resistant cell lines, and then a logistic model was used to detect gene sets (specialized gene sets of GO cellular component were downloaded from MSigDB [https://www.gsea‐msigdb.org/gsea/index.jsp and collected from references]) that were consistently associated with high or low values in the ranked lists. The *p* value < 0.05 from the logistic regression was used to determine the statistical significance of GSEA analysis.

### Metabolic Pathway Enrichment Score

The 86 metabolic pathways were downloaded from the previous paper,^[^ ^48^ ^]^ including 1660 human metabolic genes. Gene set variation analysis (GSVA, V1.34.0) was utilized to calculate the metabolic pathway enrichment score in each cell line based on transcriptomic data.

### Dimensional Reduction and Correlation Analysis

R Studio (https://rstudio.com/) was used to run custom R scripts, including Principal component analysis (PCA) for dimensional reduction of transcriptomic data, Pearson correlation analysis, Cochran–Armitage trend tests, and Student's *t*‐test. The following R packages were used: base (3.6.0), ggplot2 (3.3.5), dplyr (1.0.7), reshape2 (1.4.4), tidyverse (1.3.1), scales (1.1.1), ggsignif (0.6.2), pheatmap (1.0.12), and venn (1.10), for Graphics and statistical analysis.

### Generation of WGBS Library

For WGBS, 1 × 10^6^ cells were collected for each sample. Briefly, DNAs were extracted using Allprep DNA/RNA/Protein Mini Kit (Qiagen, 80004) following the manufacturer's instruction. The qualities of total DNAs were determined by NanoDrop 2000 and agarose electrophoresis. High‐quality DNA sample (1 µg) spiked with 26 ng unmethylated lambda DNA (Promega, D1521) was fragmented into ≈250‐bp fragments using S220 Focused‐ultrasonicator (Covairs). After end‐repairing and dA‐tailing using 5× ER/A‐Tailing Enzyme Mix (Enzymatics, Y9420L), fragmented DNAs were ligated using cytosine‐methylated barcodes and treated with bisulfite with EZ DNA Methylation‐Gold Kit (Zymo, D5006). The WGBS libraries were then generated by PCR. Subsequently, sequencing was performed in Illumina Novaseq 6000 sequencer using 150 bp pair‐end protocol.

### WGBS Data Processing and Analysis

First, WGBS sequencing reads were preprocessed with cutadapt (V1.18) to remove adaptor sequences, low‐quality bases and reads shorter than 50 bases were removed. After quality controls, the trimmed clean reads were mapped to the human reference genome (hg19) using bismark (V0.22.1) with parameters “‐N 1 –non_directional”. Then, the unmapped reads, non‐uniquely mapped reads, and PCR duplicates were removed by “deduplicate_bismark” with default parameters. Next, the methylation sites were called and the methylation percentages for individual CpG sites were calculated by the “bismark_methylation_extractor”. Only the CpG sites that were covered by at least three reads for all subsequent analyses were kept.

The methylation level in a region was computed as the average of the methylation percentages of all detected CpG sites in the region, and the regions containing at least three CpG sites were kept. The genome regions involved in this paper included promoter (defined as the 1‐kb region upstream of TSS to TSS), CpG islands (downloaded from UCSC hg19), and repeat elements (LINE, SILE and LTR, obtained from UCSC hg19).

Differentially methylated regions (DMRs) between OXPHOS inhibition sensitive and resistant cancer cells were determined by methpipe (V4.1.1). Setting the following threshold: DMRs were defined as regions containing at least three differentially methylated sites (DMCs); total methylation difference of individual DMRs was more than 20% and *p* value less than 0.01; DMRs were also identified as hypo‐ and hyper‐ methylation in OXPHOS inhibition sensitive cells. Gene annotation of DMRs was performed by “annotatePeaks.pl” from the tool HOMER (V4.1).

Integrative Genomic Viewer (IGV) was used for the visualization of DMCs and DMRs.

### DepMap Bioinformatics Analyses

For drug sensitivity analysis, processed gene expression and drug screening data (Drug sensitivity dose‐level (PRISM Repurposing Secondary Screen 19Q4) were downloaded from the DepMap Portal website. Then, the cancer cell lines were divided into four or five groups according to their drug sensitivity as indicated. Boxplot shows the NNMT/DNMT1 transcription level in each group. *P* values by Wilcox test were shown.

For gene dependency analysis of CRISPR Screen data, Genome‐scale CRISPR‐Cas9 screen result (DepMap 22Q2 Public+Score, Chronos) was obtained from the DepMap Portal website. Next, Pearson correlation coefficients between NNMT/DNMT1 transcription level and gene dependency score were performed in R Studio. Then, gene ontology (GO) enrichment analysis for the top 100 genes positively correlated with NNMT transcription and the top 100 genes negatively correlated with DNMT1 transcription in DAVID Bioinformatics Resources (v6.8) with default settings was performed, respectively. The top ten most significantly enriched items are shown in the figures.

### Data Availability

All data supporting the findings of this paper are available. The RNA‐seq and WGBS raw sequence data reported in this paper have been deposited into the Genome Sequence Archive (GSA) for humans under accession: HRA001452. RNA expression data of cancer cells were downloaded from the CCLE (https://sites.broadinstitute.org/ccle/), and DepMap (https://depmap.org/portal/). DNA methylation data of cancer cells were downloaded from CCLE. TCGA gene expression data and relevant clinical information of cancer patients were downloaded from UCSC Xena (https://xenabrowser.net/datapages/).

### Statistical Analysis

For high‐throughput sequencing, gene expression values were log2 transformed or rescale normalized to range from 0 to 10. Data analysis was performed by GraphPad Prism or R Studio. All statistical tests were comprehensively and clearly illustrated in the parallel figure legends. Two‐tailed unpaired *t*‐test or Wilcox test was used for the comparison between the two groups. Mann–Whitney test was used for the comparison between groups of more than two. Pearson test and Cochran–Armitage trend test were used for correlation analysis. For comparisons, *p* < 0.05 was presented as statistical significance.

All other data were represented as mean ± SD. Statistical analysis was performed using one or two‐way ANOVA, Mann–Whiney test, or paired Student's *t*‐test. All calculations were performed using GraphPad Prism 9.1.2 (GraphPad Software, CA, USA).

## Conflict of Interest

The authors declare no conflict of interest.

## Author Contributions

C.W., Y.L., W.L., and T.Z. contributed equally to this work. Performed and analyzed the mouse experiments: C.W. and S.L. Performed and analyzed experiments showing a synergistic effect of NNMT and DNMT1: Y.L. Performed and analyzed bioinformatic analysis: W.L. Performed and analyzed experiments with patient samples: C.W., S.L., and T.Z. Provided technical support: C.Z., L.H., J.C., and L.F. Provided cell lines and constructive advice: P.W., L.F., H.Y., and J.C. Supervised: J.F. and C.J. Prepared patient samples: J. F. and L.H. Performed bioinformatic analysis: C.J. and W.L. Conceived and supervised the entire study and wrote the manuscript with input from J.C., C.J., C.W, Y.L. T.H., and other authors: Y.S.

## Supporting information

Supporting Information

Supplemental Table 1

## Data Availability

The data that support the findings of this study are openly available in Genome Sequence Archive (GSA) for human at https://ngdc.cncb.ac.cn/gsa‐human/, reference number 1452.
