# Supplementary material for: NNMT‐DNMT1 Axis is Essential for Maintaining Cancer Cell Sensitivity to Oxidative Phosphorylation Inhibition
Source: Adv Sci (Weinh). 2022 Nov 16;10(1):2202642. doi: 10.1002/advs.202202642 (PMC9811437; doi:10.1002/advs.202202642)
Supplement: Supplementary file 1 — Supporting Information [file ADVS-10-2202642-s001.pdf]

ADVANCED SCIENCE
Open Access

# Supporting Information

for *Adv. Sci.*, DOI 10.1002/advs.202202642

NNMT-DNMT1 Axis is Essential for Maintaining Cancer Cell Sensitivity to Oxidative Phosphorylation Inhibition

*Changqing Wu, Yu'e Liu, Wenju Liu, Tianhui Zou, Shaojuan Lu, Chengjie Zhu, Le He, Jie Chen, Lan Fang, Lin Zou, Ping Wang, Lihong Fan, Hongxiang Wang, Han You, Juxiang Chen*, Jing-Yuan Fang*, Cizhong Jiang* and Yufeng Shi**

ADVANCED SCIENCE
Open Access

Supporting Information

# NNMT-DNMT1 axis is essential for maintaining cancer cell sensitivity to oxidative phosphorylation inhibition

*Changqing Wu[1,9], Yu'e Liu[1,9], Wenju Liu[2,9], Tianhui Zou[3,9], Shaojuan Lu[1], Chengjie Zhu[1], Le He[1], Jie Chen[1], Lan Fang[1], Lin Zou[4], Ping Wang[1], Lihong Fan[5], Hongxiang Wang[6], Han You[8], Juxiang Chen[6,*], Jing-Yuan Fang[3,*], Cizhong Jiang[2,*], Yufeng Shi[1,2,10,*]*

Supplementary Materials for

# NNMT-DNMT1 axis is essential for maintaining cancer cell sensitivity to oxidative phosphorylation inhibition

Changqing Wu[1,9], Yu'e Liu[1,9], Wenju Liu[2,9], Tianhui Zou[3,9], Shaojuan Lu[1], Chengjie Zhu[1], Le He[1], Jie Chen[1], Lan Fang[1], Lin Zou[4], Ping Wang[1], Lihong Fan[5], Hongxiang Wang[6], Han You[8], Juxiang Chen[6,]*, Jing-Yuan Fang[3,]*, Cizhong Jiang[2,]*, Yufeng Shi[1,2,10,]*

* **Correspondence:** J.C (juxiangchen@smmu.edu.cn), J.F. (jingyuanfang@sjtu.edu.cn), C.J. (czjiang@tongji.edu.cn) and Y.S. (yshi@tongji.edu.cn).

## The PDF file includes:

Supplementary Figure1 to 6

Captions for Supplementary Figure 1 to 6

Supplementary Table 1

**Supplementary Figure 1**

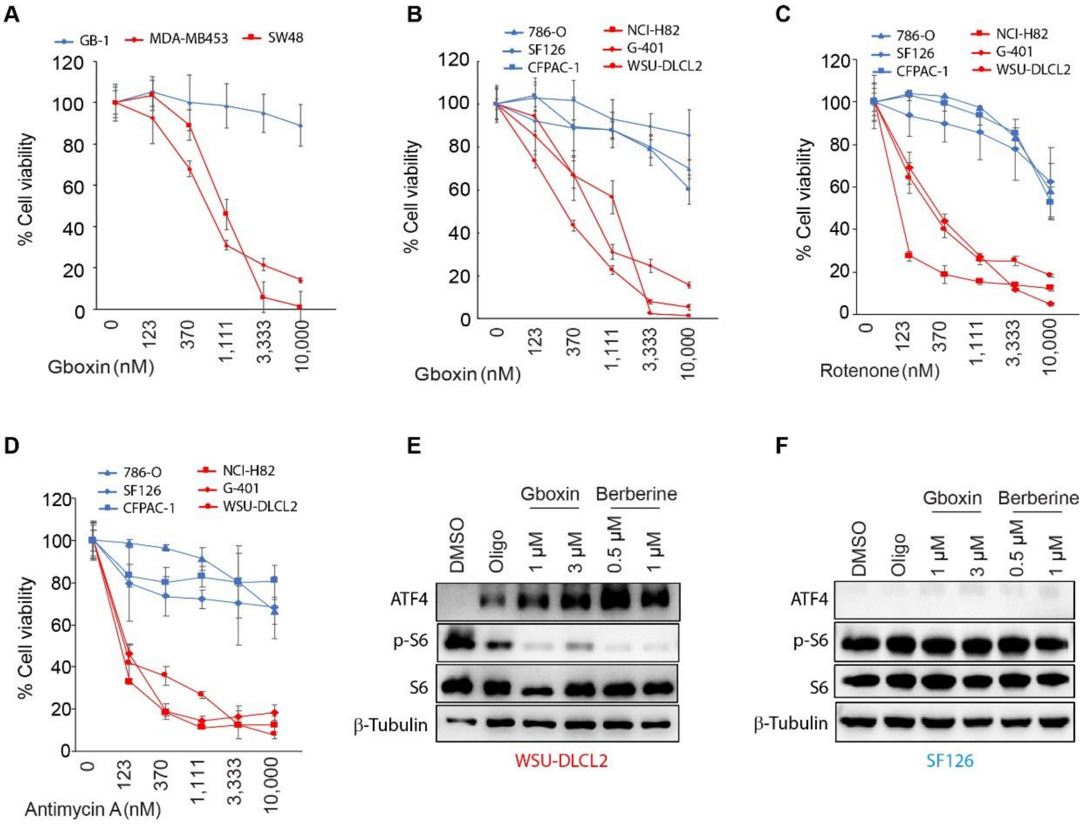


## Figure S1

## Gboxin-sensitive and resistant cancer cell lines are also sensitive or resistant to other OXPHOS inhibitors.

A) and B), Cell viability analysis with CellTilter Glo® reagent shows. A) MDA-MB453 and SW48 (red lines) are Gboxin-sensitive cancer cell lines while GB-1 (blue line) is a resistant one; B) Cancer cell lines (NCI-H82, G-401, WSU-DLCL2) are sensitive while cancer cell lines (786-O, SF126, CFPAC-1) are resistant to Gboxin treatment when all cells were cultured and treated in RMPI1640 medium. After cancer cells as indicated incubated with a series dilution of Gboxin for 3 days, cell viability analysis was performed. Mean ± SD. n = 3. C) and D), Cell viability analysis shows cancer cell lines (NCI-H82, G-401, WSU-DLCL2) are sensitive (red lines) while cancer cell lines (786-O, SF126, CFPAC-1) are resistant (blue lines) to treatments of Rotenone (OXPHOS complex I inhibitor, C) and Antimycin A (OXPHOS complex III inhibitor, D). After cancer cells as indicated were incubated with a series of dilutions of Rotenone (C) and Antimycin A (D) for 3 days, cell viability analysis was performed. Mean ± SD. n = 3. E) and F), Treatments of Oligomycin, Gboxin and Berberine induce ATF4 while suppressing p-S6 expression in WSU-DLCL2 (E) but not in SF126 (F) cancer cells. After cancer cells as indicated were treated with Oligomycin (1 μM), Gboxin (1 μM and 3 μM) or Berberine (0.5 μM and 1 μM) for 6 hours, Western blot assays were applied for quantitative analysis of ATF4 and p-S6

expression.

Supplementary Figure 2

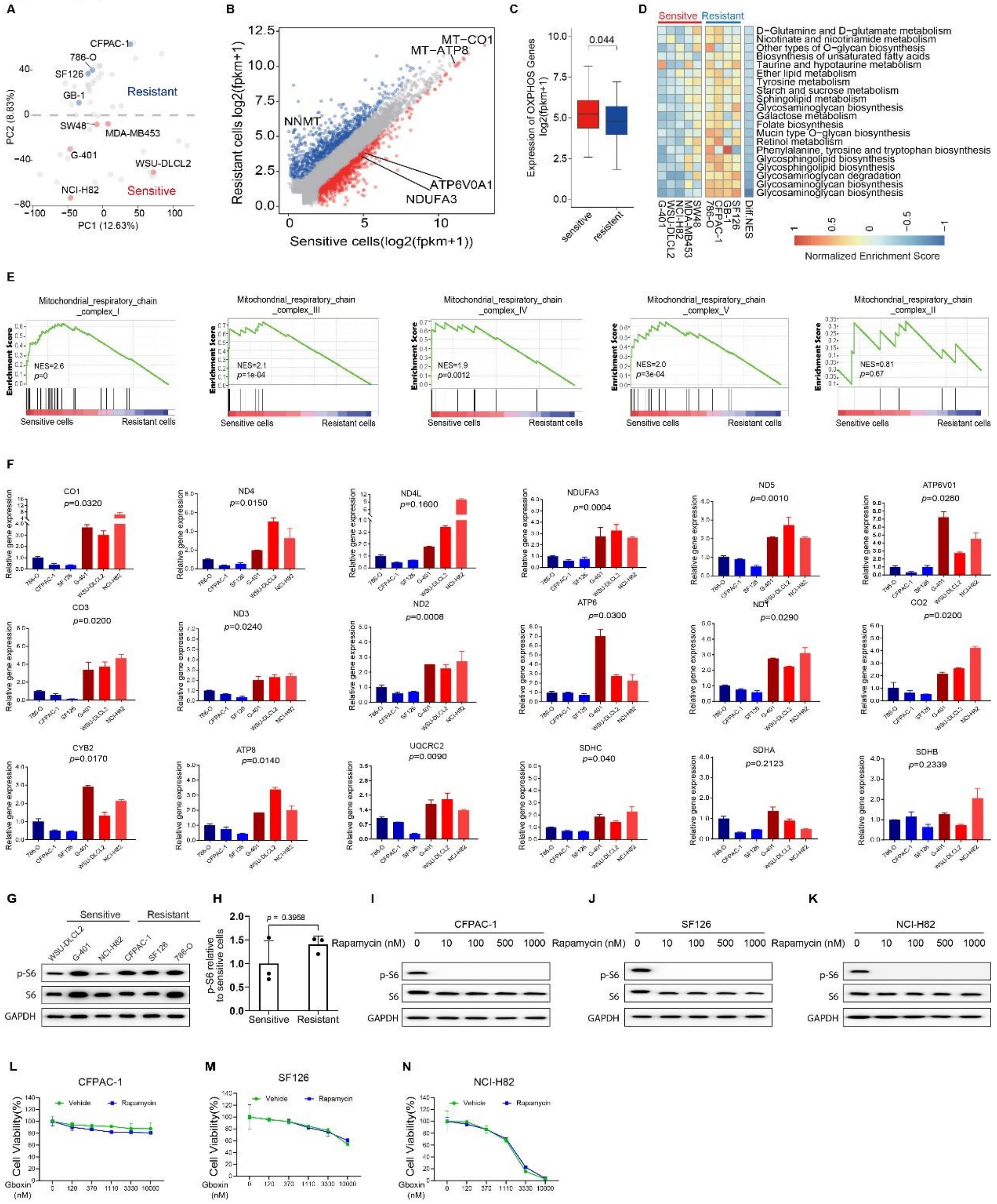


# Figure S2

## OXPHOS inhibition-sensitive cancer cell lines showed enhanced mitochondrial OXPHOS expression compared with resistant ones.

A), Principal component analysis (PCA) with the whole transcriptome of OXPHOS-inhibitor sensitive (NCI-H82, G-401, MDA-MB-453, WSU-DLCL2 and SW48) and resistant (786-O, SF126, CFPAC-1 and GB-1) cell lines reveals distinct gene

transcription pattern between these two groups of cancer cell lines. Red and blue dots represent OXPHOS sensitive and resistant cell lines, respectively. Data from DepMap database. B), Scatter plot reveals a significant portion of genes are differentially expressed genes (DEGs) between OXPHOS sensitive (NCI-H82, G-401, MDA-MB-453, WSU-DLCL2 and SW48) and resistant (786-O, SF126, CFPAC-1 and GB-1) cancer cell lines. The whole Genome transcriptome was analyzed and presented. NNMT expression is greatly downregulated while multiple mitochondria metabolic-related genes, such as MT-CO1, MT-ATP8, ATPV0A1 and NDUFA3, are upregulated in OXPHOS sensitive cell lines compared with resistant ones. Red and blue dots represent significantly upregulated and downregulated DEGs whose expression changed by at least two folds in OXPHOS inhibition sensitive cancer cell lines compared with resistant ones, respectively. Blue dots are significantly downregulated DEGs in sensitive cancer cell lines, red dots significantly upregulated DEGs. C), Box plots show the average expression of OXPHOS genes in OXPHOS-inhibitor sensitive (NCI-H82, G-401, MDA-MB-453, WSU-DLCL2 and SW48) and resistant (786-O, SF126, CFPAC-1 and GB-1) cell lines. Paired *t*-test. D), Heatmap shows downregulated metabolic pathways (blue) in OXPHOS inhibition sensitive cancer cell lines compared with those in resistant cancer cell lines. Diff NES: difference of normalized enrichment score (sensitive - resistant). Gene set variation analysis (GSVA) is performed based on metabolic related pathways. E), Gene set enrichment analyses (GSEA) show that genes functioning in mitochondrial OXPHOS complexes I, III, IV, and V, but not complex II are significantly upregulated in OXPHOS sensitive cancer cells compared with those in the resistant ones. F), Confirmation enhanced mitochondria-related genes (CO1, ND4, ND4L, NDUFA3, ND5, ATP6V01, CO3, ND3, ND2, ATP6, ND1, CO2, CYB, ATP8, UQCRC2, SDHC, SDHA and SDHB in OXPHOS inhibition sensitive cell lines (G-401, WSU-DLCL2, NCI-H82 and MDA-MB-453) compared with those in resistant ones (786-O, CFPAC-1 and SF126) using real-time quantitative PCR. Mean ± SD. n = 3. Paired *t*-test. G) and H), Western blot shows the expression of p-S6 in OXPHOS inhibition sensitive (WSU-DLCL2, G-401 and NCI-H82) and resistant (CFPAC-1, SF126 and 786-O) cancer cell lines. H) Quantification of (G) by image J software. Paired *t*-test. I-K), Rapamycin treatment inhibits p-S6 expression in OXPHOS inhibition sensitive (NCI-H82) and resistant (CFPAC-1 and SF126) cancer cell lines. After cancer cells as indicated were treated with rapamycin (10 nM,100 nM, 500 nM and 1000 nM) for 6 hours, western blot assays were applied to detect p-S6 expression. n = 2 at least. L-N), Cell viability analysis shows OXPHOS inhibition sensitive or resistant cancer cell lines are still sensitive or resistant to Gboxin, respectively, when p-S6 was inhibited. After cancer cells as indicated were incubated with 100 nM rapamycin and a series of dilutions of Gboxin for 3 days, cell viability analysis was performed. Mean ± SD. n = 3.

Supplementary Figure 3

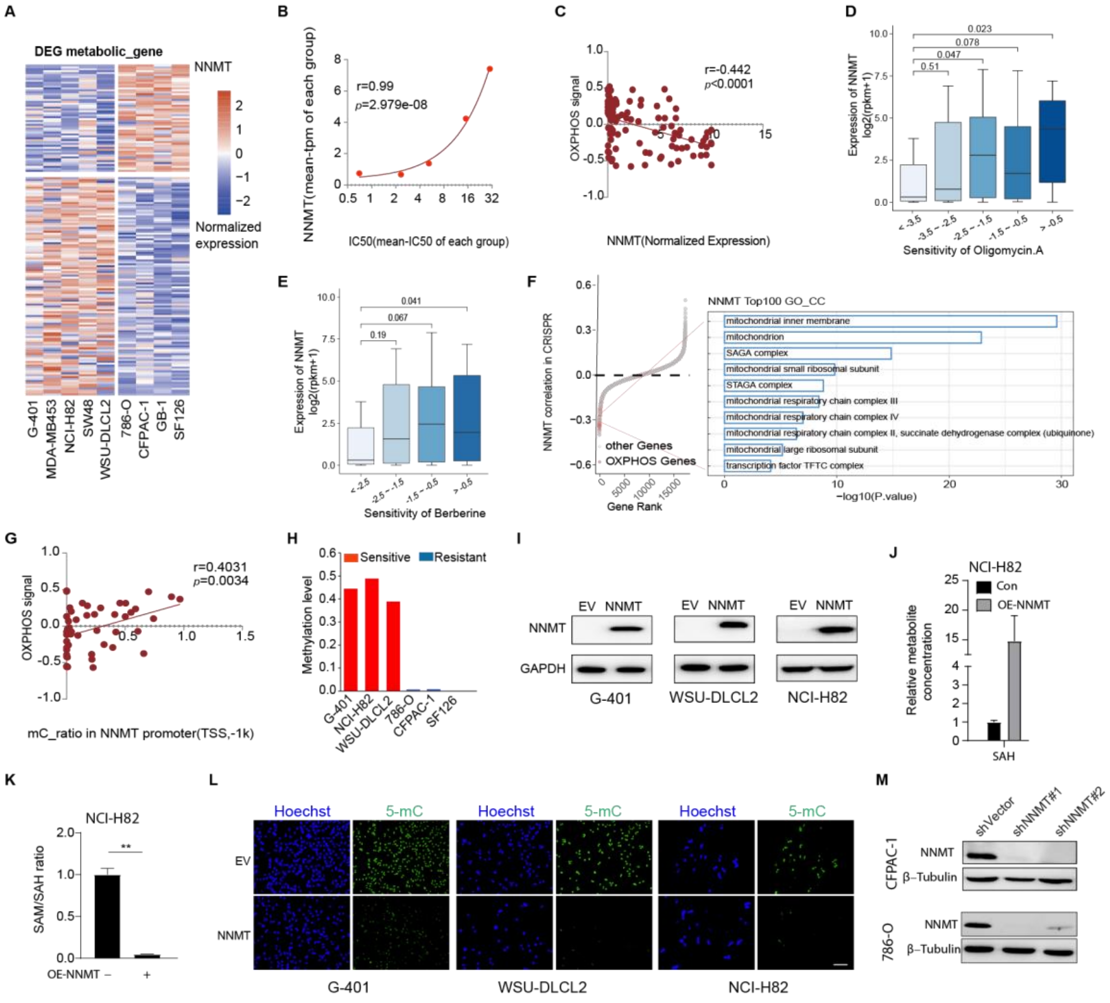


# Figure S3

## NNMT expression is negatively correlated with cancer cell sensitivity to OXPHOS inhibition.

A), Heatmap shows NNMT is one of the differentially expressed metabolic-related genes in OXPHOS inhibition-sensitive cancer cell lines (NCI-H82, G-401, MDA-MB-453, WSU-DLCL2 and SW48) compared with those in resistant ones (786-O, SF126, CFPAC-1 and GB-1). Red and blue colors represent high and low expression, respectively. B), the Cochran-Armitage trend test reveals a strong positive (negative) correlation between NNMT expression and Gboxin IC50s (sensitivity). The IC50 value is the mean of IC50 values for cancer cell lines in each group as in Figure 3B. So is NNMT expression level. C), NNMT expression level is negatively correlated with OXPHOS activity in the 57 cancer cell lines used in this study. Expression data of the NNMT gene was extracted from CCLE, DEMAP and our own data. Pearson test used. D and E), Boxplot shows the NNMT transcription negatively correlated with cancer cell sensitivity to Oligomycin (D) or

Berberine (E) treatment. NNMT expression level, Oligomycin and Berberine sensitivity were downloaded from the DepMap database. Oligomycin and Berberine sensitivity were divided into 5 or 4 groups according to their sensitivity as indicated. P values by the wilcox.test are shown. F), Scatter plot shows NNMT expression negatively correlated with the dependency of OXPHOS genes in cancer cell lines. The genes dependency score was downloaded from the CRISPR-Cas9 screen result in the DepMap database. GO cellular component analysis of top 100 positively correlated genes is performed. G), DNA methylation level in NNMT promoter (from -1000 bp to TSS) and OXPHOS pathway signal in the 57 cancer cell lines used in this study. Methylation data of NNMT promoter were from CCLE and our own data. (Pearson test used. H), The histogram shows the DNA methylation level in the DMR in the NNMT promoter (dotted box in Figure 3G). I), Western blot shows overexpression of NNMT in the OXPHOS inhibition-sensitive cancer cell lines (G-401, WSU-DLCL2 and NCI-H82). J) and K), NNMT overexpression increases intracellular SAH levels and reduces the SAM/SAH ratio. Intracellular SAH levels were normalized by total protein in indicated cell lines. n=3. L), Representative images of global 5-mythylated cytosine in NNMT upregulated cancer cells (G-401, WSU-DLCL2 and NCI-H82) using immunofluorescence staining. EV, empty vector. 5-mC, 5-mythylated cytosine. n > 5. M), Western blot shows knockdown of NNMT in the OXPHOS inhibition resistant cancer cell line CFPAC-1 and 786-O.

**Supplementary Figure 4**

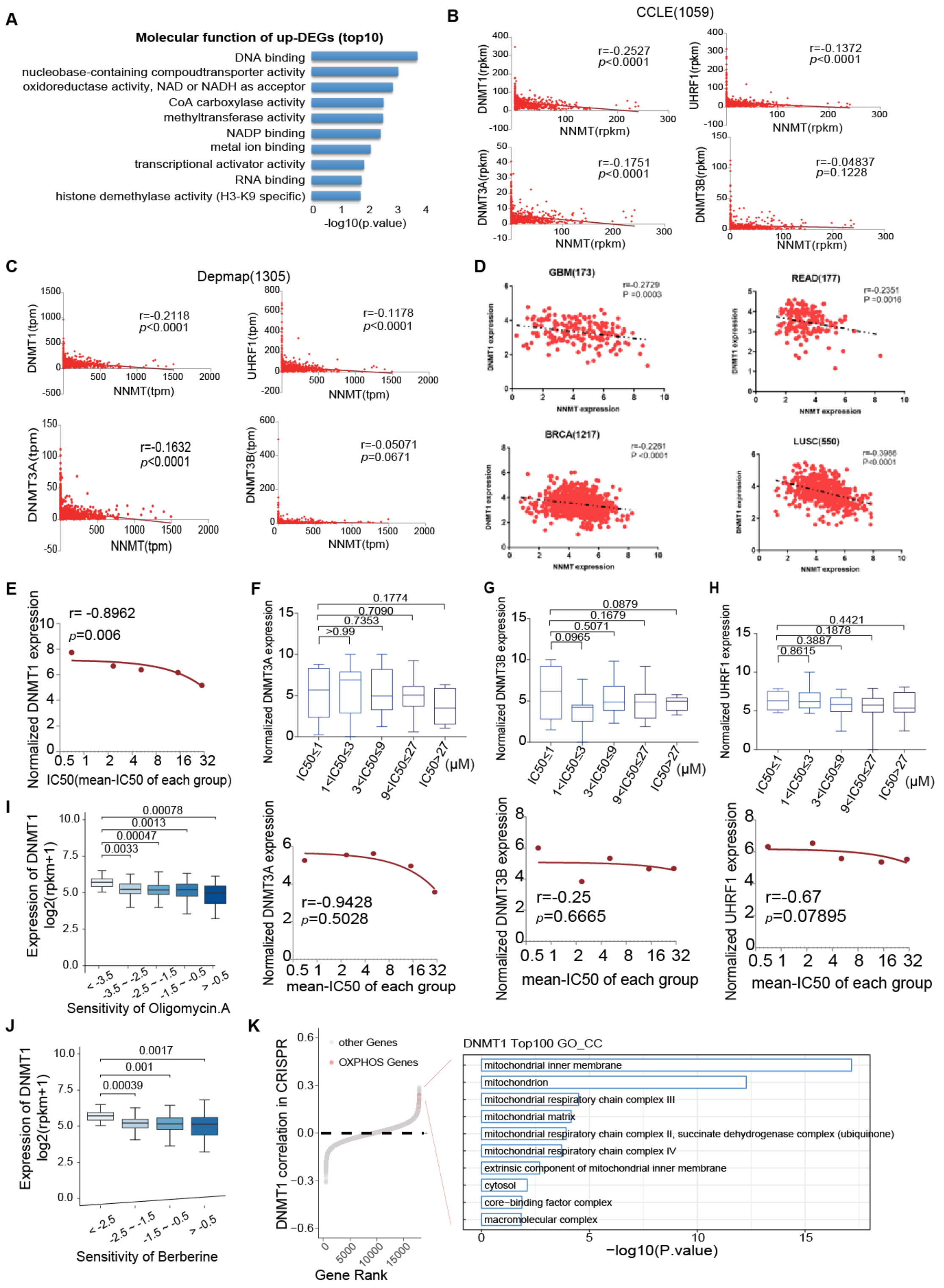


# Figure S4

**DNMT1 expression positively correlated with cancer cell sensitivity to OXPHOS inhibition.**

A), GO molecular function enrichment analysis of the genes up-regulated in OXPHOS inhibition sensitive cancer cells (NCI-H82, G-401, MDA-MB-453, WSU-DLCL2 and SW48)

compared with that in the resistant cancer cells (786-O, SF126, CFPAC-1 and GB-1) are enriched on pathways including DNA binding, metabolism related, methyltransferase *et*. *al*. B), Correlation analysis showed a significant negative correlation of NNMT expression with DNMT1, UHRF1 and DNMT3A, but not DNMT3B with transcription data for 1,059 cell lines in CCLE database. Student's t-test was used. C), Correlation analysis showed a significant negative correlation of NNMT expression with DNMT1, UHRF1 and DNMT3A, but not DNMT3B with transcription data for 1,305 cell lines in the Depmap database. Student's *t*-test was used. D), Correlation analysis showed a significant negative correlation between NNMT and DNMT1 expression in 173 glioblastoma multiforme (GBM), 177 rectum adenocarcinomas (READ), 1,104 breast invasive carcinomas (BRCA) and 550 lung squamous cell carcinomas (LUSC). Transcriptional Data are from TCGA. Student's t-test used. E), the Cochran-Armitage trend test reveals strong positive (negative) correlation between DNMT1 expression and Gboxin sensitivity (IC50s) with data used in (Figure 3B). Pearson correlation coefficient is indicated. F)-H), Left panel: Boxplots show no significant difference to DNMT3A (F), DNMT3B (G) and UHRF1 (H) expression between Gboxin sensitive and resistant cancer cell lines, and right panel: there is no significant correlation between DNMT3A (F), DNMT3B (G) and UHRF1 (H) expression and Gboxin IC50s (sensitivity) with data used in the left panel. 57 cancer cell lines in Gboxin sensitivity screen are divided into 5 groups according to their Gboxin sensitivity as indicated. Transcription data of DNMT3A, DNMT3B and UHRF1 for 46 out of 57 cancer lines was extracted from the Depmap and CCLE, and the expression value of DNMT3A, DNMT3B and UHRF1 is rescaled to range from 0 to 10. The Mann-Whitney test (left panel) and the Cochran-Armitage trend test (right panel) were used. Pearson correlation coefficient is indicated. I) and J), Boxplot shows DNMT1 transcription positively correlated with cancer cell sensitivity to Oligomycin (I) or Berberine (J) treatment. DNMT1 expression level, Oligomycin and Berberine sensitivity were downloaded from DepMap database. Oligomycin and Berberine sensitivity were divided into 5 or 4 groups according to their sensitivity as indicated. The P values by the Wilcox.test are shown. K), Scatter plot shows DNMT1 expression positively correlated with the dependency of OXPHOS genes in cancer cell lines. The genes dependency score was downloaded from the CRISPR-Cas9 screen result in the DepMap database. GO cellular component analysis of the top 100 negatively correlated genes is performed.

Supplementary Figure 5

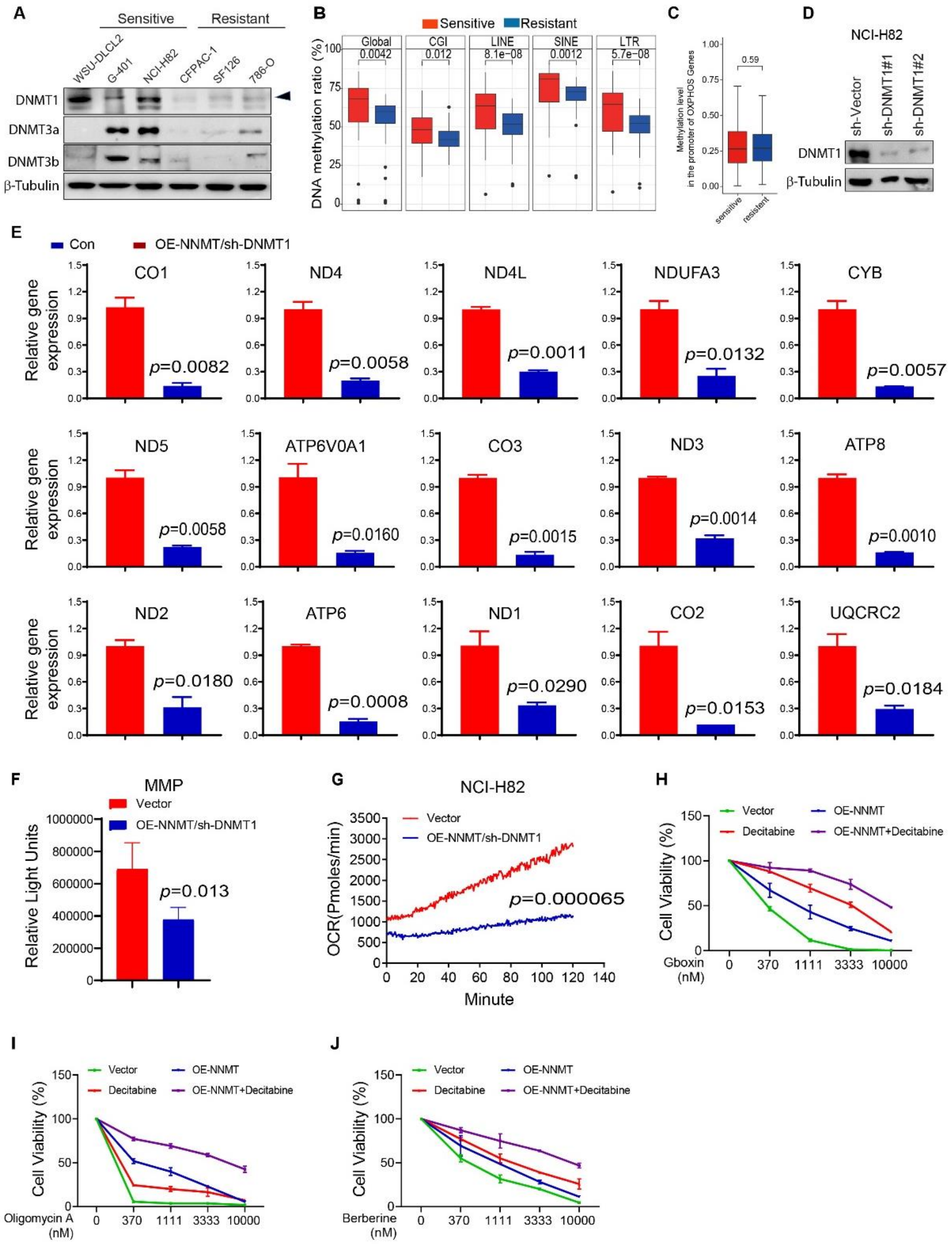


# Figure S5

**NNMT overexpression and Decitabine treatment synergistically reduce cancer cell sensitivity to OXPHOS inhibition.**

A), Representative western blot shows expression of DNMT1, DNMT3A, DNMT3B in OXPHOS inhibition-sensitive (WSU-DLCL2, G-401, and NCI-H82) and resistant (786-O, SF126, and CFPAC-1) cancer cell lines. B), Box plots showed higher genomic DNA

methylation levels in OXPHOS inhibition sensitive cancer cell lines (red) compared with those in the resistant ones (blue). Global genome, CpG island (CGI), long interspersed nuclear elements (LINE), short interspersed nuclear elements (SINE) and long terminal repeat elements (LTR). OXPHOS inhibition sensitive cancer cell lines: NCI-H82, G-401, MDA-MB-453, WSU-DLCL2 and SW48; OXPHOS inhibition resistant cancer cell lines: 786-O, SF126, CFPAC-1 and GB-1. Sensitive and resistant cancer cell lines were treated with bisulfite, genomic DNA methylation was sequenced. The methylation level in a region was computed as the average of the methylation percentages of all detected CpG sites in the region. The methylation levels of regions located on the same chromosome were averaged for plotting. Paired *t*-test. C), Box plots showed no significant change of methylated OXPHOS genes between OXPHOS inhibition sensitive (NCI-H82, G-401, MDA-MB-453, WSU-DLCL2 and SW48) and resistant (786-O, SF126, CFPAC-1 and GB-1) cancer cell lines. Paired *t*-test. D), Representative western blot confirms DNMT1 knockdown in NCI-H82 cells after infection of lentivirus carrying DNMT1 targeting shRNA#1 or #2 for 10 days. E), Confirmation of downregulated mitochondria-related genes (CO1, ND4, ND4L, NDUFA3, CYB, ND5, ATP6V01, CO3, ND3, ATP8, ND2, ATP6, ND1, CO2 and UQCRC2) in OE-NNMT/sh-DNMT1 NCI-H82 cell line using real-time quantitative PCR. Mean ± SD. n = 3. Paired *t*-test. F), Decreased MMP in OE-NNMT/sh-DNMT1 NCI-H82 cell line. TMRM (200nM) incubated with indicated cancer cell lines for 30 min at 37℃. Data were collected by flow cytometry. Paired *t*-test. G), Long-term OCR determination shows compromised OCR in OE-NNMT/sh-DNMT1 NCI-H82 cell line. n > 2. Paired *t*-test was used to show the significant change of increased OCR at 120 minutes. H)-J), NNMT overexpression and Decitabine treatment exhibit synergistic effects on reducing G-401 sensitivity to Gboxin (H), Oligomycin (I) and Berberine (J). G-401 cells with NNMT overexpression, Decitabine treatment or both are treated with a series of dilutions of Gboxin, Oligomycin, and Berberine for 3 days. Cell viability is then measured by CellTilter Glo® reagent. Mean± SD. n = 3.

**Supplementary Figure 6**

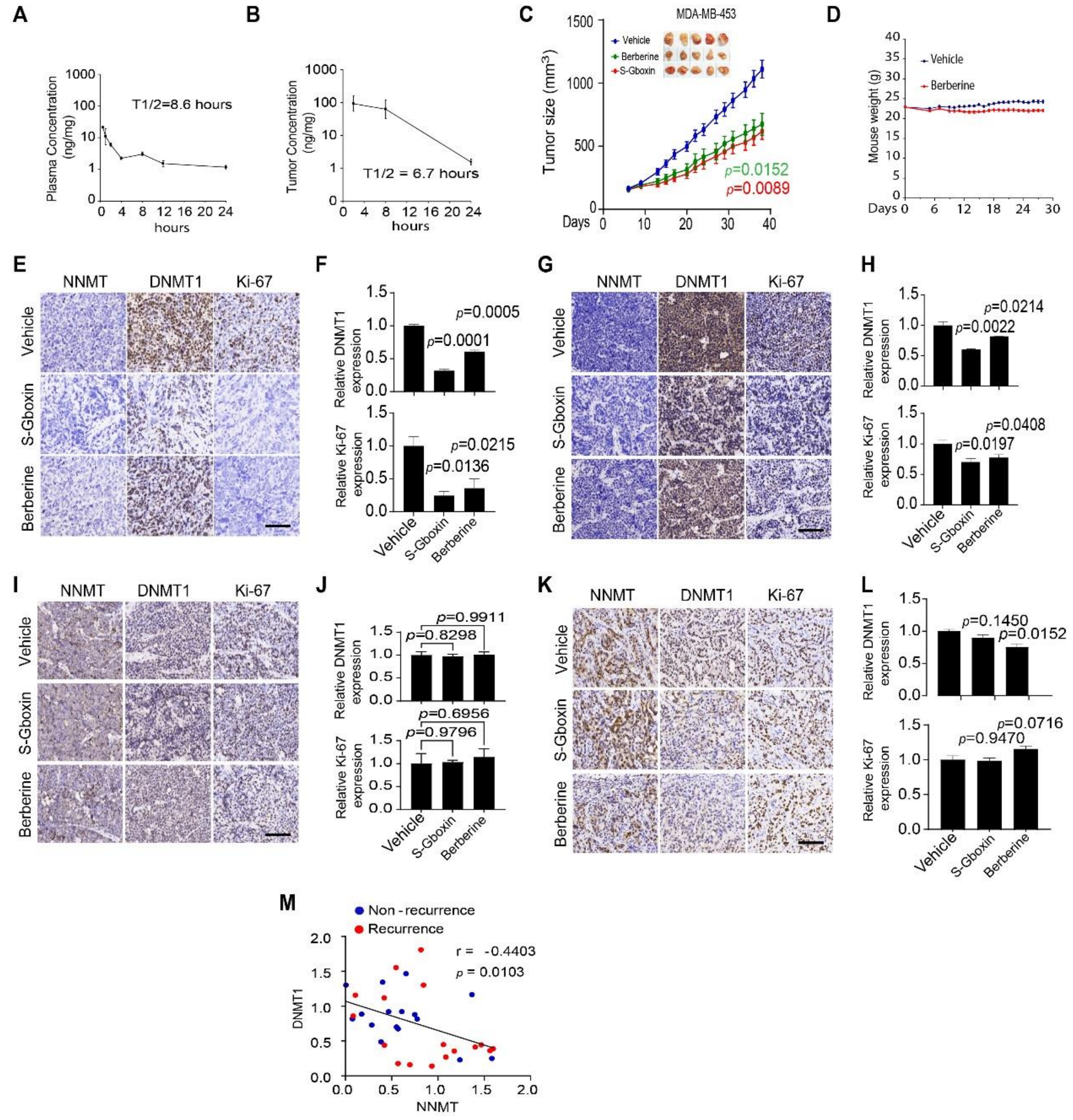


# Figure S6

**OXPHOS inhibitors, S-Gboxin and Berberine, inhibit the growth of xenograft tumors by OXPHOS sensitive cells but not resistant ones; and negative correlation between NNMT and DNMT1 expression in 32 CRAs in patients.**

A), Berberine plasma half-life. n = 3. B), Berberine tumor pharmacokinetic (PK) data. C), $5x10^6$ MDA-MB-453 (sensitive to OXPHOS inhibition) were subcutaneously injected into flanks of nude mice. 5 days after the transplant, mice were administrated intraperitoneally with S-Gboxin (10mg/kg/day) or Berberine (10mg/kg/day). Tumor growth was assessed every 2-3 days and calculated with the formula (1/2 * length * $width^2$). Tumor n=9. Images show five tumors on the day all mice were sacrificed after the treatments as indicated. D), Mice treated with Berberine at 10 mg/kg/day for a 26-day period exhibit slight weight loss compared with vehicle-treated mice. Vehicle (n=8); Berberine (n=8). E)-L), Representative

Immunostaining images and corresponding quantification for NNMT, DNMT1 and Ki-67 expression with NCI-H82 (E and F), MDA-MB-453 (G-H), NCI-H82-OE-NNMT/sh-DNMT1 (I-J) and CFPAC-1 (K-L) tumors as in figure 5E, figure 5F and figure 5G, respectively. F) quantification of (E); H) quantification of (G);(J) quantification of (I); and L) quantification of (K). Scale bar, 100 uM. Paired *t*-test. M), Scatter plot shows the negative correlation between NNMT and DNMT1 expression in 32 CRAs as described in (Figure 6E, 6F). The correlation value was calculated by the Pearson correlation coefficient method and p value was calculated by Two-tailed Pearson’s Correlation.
