# Supplementary material for: NNMT‐DNMT1 Axis is Essential for Maintaining Cancer Cell Sensitivity to Oxidative Phosphorylation Inhibition
Source: Adv Sci (Weinh). 2022 Nov 16;10(1):2202642. doi: 10.1002/advs.202202642 (PMC9811437; doi:10.1002/advs.202202642)
Supplement: Supplementary file 2 — Supplemental Table 1 [file ADVS-10-2202642-s002.pdf]

ADVANCED SCIENCE
Open Access

Supporting Information

for *Adv. Sci.*, DOI 10.1002/advs.202202642

NNMT-DNMT1 Axis is Essential for Maintaining Cancer Cell Sensitivity to Oxidative Phosphorylation Inhibition

Changqing Wu, Yu'e Liu, Wenju Liu, Tianhui Zou, Shaojuan Lu, Chengjie Zhu, Le He, Jie Chen, Lan Fang, Lin Zou, Ping Wang, Lihong Fan, Hongxiang Wang, Han You, Juxiang Chen, Jing-Yuan Fang*, Cizhong Jiang* and Yufeng Shi**

Table S1

Section I (Basic Information)				doubling time (hr)
AM-38	JCRB	IFO50492	brain, glioblastoma	23
GB-1	JCRB	IFO50489	brain, glioblastoma	28
U-87 MG	ATCC	HTB-14	brain, glioblastoma	36
U-251 MG	ECACC	9063001	brain, glioblastoma as	34
SF126	JCRB	IFO50286	brain, glioblastoma m	30
D283 Med	ATCC	HTB-185	brain, cerebellum med	32
Daoy	ATCC	HTB-186	brain, desmoplastic ce	41
HCC70	ATCC	CRL-2315	breast, primary ductal	36
MDA-MB-231	ATCC	HTB-26	breast, adenocarcinon	31
BT-549	ATCC	HTB-122	breast, ductal carcinoi	31
HCC1806	ATCC	CRL-2335	breast, primary acanth	21
MDA-MB-453	ATCC	HTB-131	breast, metastatic carc	34
COLO 320HSR	ATCC	CCL-220.1	colon, colorectal aden	23
SW48	ATCC	CCL-231	colon, colorectal aden	25
Gp2D	ECACC	95090714	colon, colorectal aden	25
LS 180	ATCC	CL-187	colon, colorectal aden	28
769-P	ATCC	CRL-1933	kidney, renal cell aden	25
786-O	ATCC	CRL-1932	kidney, renal cell aden	28
G-401	ATCC	CRL-1441	kidney, rhabdoid tumo	24
G-402	ATCC	CRL-1440	kidney, renal leiomyob	28
ACHN	ATCC	CRL-1611	kidney, renal cell aden	31
OCI-AML-4	DSMZ	ACC-729	leukemia, acute myeloid leukemia	19
OCI-AML-5	DSMZ	ACC-247	leukemia, acute myeloid leukemia	18
OCI-M1	DSMZ	ACC-529	leukemia, acute myeloid leukemia	25
OCI-AML-3	DSMZ	ACC-582	leukemia, acute myeloid leukemia	17
Jurkat, Clone	ATCC	TIB-152	leukemia, acute T cell,	16
ATN-1	RIKEN	RBRC-RCB144	leukemia, adult T-cell,	34
CCRF-CEM	ATCC	CCL-119	leukemia, acute T lymp	29
SNU-398	ATCC	CRL-2233	liver, hepatocellular ca	29
SNU-449	ATCC	CRL-2234	liver, hepatocellular ca	36
C3A	ATCC	CRL-10741	liver, hepatocellular ca	23

HCC15	KCLB	70015	lung, non small cell lu	39
NCI-H1155	ATCC	CRL-5818	lung, non-samll cell lu	35
NCI-H1651	ATCC	CRL-5884	lung, non-small cell lu	40
NCI-H1793	ATCC	CRL-5896	lung, non-small cell lu	28
NCI-H1048	ATCC	CRL-5853	lung, small cell lung ca	35
NCI-H196	ATCC	CRL-5823	lung, small cell lung ca	34
NCI-H82	ATCC	HTB-175	lung, small cell lung ca	33
NCI-H2081	ATCC	CRL-5920	lung, small cell lung ca	37
U-937	ATCC	CRL-1593.2	lymphoma, hitiocytic	22
SU-DHL-2	ATCC	CRL-2956	lymphoma, B cell lymphoma	25
WSU-DLCL2	DSMZ	ACC-575	lymphoma, B cell lymphoma	16
OCI-LY-19	DSMZ	ACC-528	lymphoma, B cell lymphoma	34
HH	ATCC	CRL-2105	lymphoma, cutaneous	33
MJ	ATCC	CRL-8294	lymphoma, cutaneous	25
**H9**	ATCC	HTB-176	lymphoma, cutaneous	33
PANC-1	ATCC	CRL-1469	pancreas, ductal carci	49
SU.86.86	ATCC	CRL-1837	pancreas, ductal carci	44
MIA PaCa-2	ATCC	CRL-1420	pancreas, pancreatic C	21
CFPAC-1	ATCC	CRL-1918	pancreas, ductal aden	29
PC-3	ATCC	CRL-1435	prostate, adenocarcin	31
22RV1	ATCC	CRL-2505	prostate, human carci	34
DU 145	ATCC	HTB-81	prostate, carcinoma	38
A-375	ATCC	CRL-1619	skin, malignant melan	21
CHL-1	ATCC	CRL-9446	skin, malignant melan	24
G-361	ATCC	CRL-1424	skin, malignant melan	32
GAK	JCRB	JCRB0180	skin, malignant melan	28
